# Tomato transcriptome and mutant analyses suggest a role for plant stress hormones in the interaction between fruit and *Botrytis cinerea*

**DOI:** 10.3389/fpls.2013.00142

**Published:** 2013-05-14

**Authors:** Barbara Blanco-Ulate, Estefania Vincenti, Ann L. T. Powell, Dario Cantu

**Affiliations:** ^1^Department of Plant Sciences, University of California, DavisDavis, CA, USA; ^2^Department of Viticulture and Enology, University of California, DavisDavis, CA, USA

**Keywords:** plant-pathogen, ripening, resistance, susceptibility, ethylene, salicylic acid, jasmonic acid, microarray

## Abstract

Fruit–pathogen interactions are a valuable biological system to study the role of plant development in the transition from resistance to susceptibility. In general, unripe fruit are resistant to pathogen infection but become increasingly more susceptible as they ripen. During ripening, fruit undergo significant physiological and biochemical changes that are coordinated by complex regulatory and hormonal signaling networks. The interplay between multiple plant stress hormones in the interaction between plant vegetative tissues and microbial pathogens has been documented extensively, but the relevance of these hormones during infections of fruit is unclear. In this work, we analyzed a transcriptome study of tomato fruit infected with *Botrytis cinerea* in order to profile the expression of genes for the biosynthesis, modification and signal transduction of ethylene (ET), salicylic acid (SA), jasmonic acid (JA), and abscisic acid (ABA), hormones that may be not only involved in ripening, but also in fruit interactions with pathogens. The changes in relative expression of key genes during infection and assays of susceptibility of fruit with impaired synthesis or perception of these hormones were used to formulate hypotheses regarding the involvement of these regulators in the outcome of the tomato fruit–*B. cinerea* interaction.

## Introduction

Disease resistance or susceptibility of a plant depends not only on the specific plant–pathogen combination, but also on the developmental stage of the host tissues. The ripening process of fleshy fruit is an example of a developmental transition that coincides with increased susceptibility to pathogens. Ripening involves a complex network of regulatory and hormone-mediated pathways leading to significant changes in the physiological and biochemical properties of the fruit (Giovannoni, 2004). Among the ripening events, modifications in cell wall structure and composition, conversion of starch into simple sugars, changes in apoplastic pH and redox state, and decline in the concentration of antimicrobial metabolites contribute to susceptibility of fruit to pathogens (Prusky and Lichter, 2007; Cantu et al., 2008a,b). The enhanced susceptibility of ripe fruit to pathogens could be a default outcome of ripening or, alternatively, could be promoted by some, but not all, ripening processes (Cantu et al., 2009).

Fruit pathogens exhibit necrotrophic, biotrophic, or hemibiotrophic lifestyles (Prusky and Lichter, 2007; Cantu et al., 2008b), categories that reflect different infection strategies (Glazebrook, 2005). Necrotrophs, such as the ascomycete, *Botrytis cinerea*, cause necrosis by deploying hydrolytic enzymes (Van Kan, 2006), secreting toxins (Govrin et al., 2006; Dalmais et al., 2011) and/or hijacking the plant's enzymatic machinery (Cantu et al., 2009). Biotrophs depend on the integrity of plant host tissues and have developed strategies to deceive the host to obtain nutrients without inducing plant defenses or cell death (Perfect et al., 1999; Glazebrook, 2005). Hemibiotrophs are those pathogens that switch lifestyles at different developmental phases and/or in certain environmental conditions (Glazebrook, 2005; Kleemann et al., 2012). Therefore, the infection strategies of different pathogens challenge the competency of the plant host to respond and deploy effective defense mechanisms.

Tomato (*Solanum lycopersicum*) has served as a model organism to study fruit ripening (Giovannoni, 2004) and has emerged as an informative experimental system to characterize the molecular regulation of the ripening-related susceptibility to pathogens, in particular to necrotrophic fungi, such as *B. cinerea* (Powell et al., 2000; Flors et al., 2007; Cantu et al., 2008a, 2009). *B. cinerea* fails to develop in unripe (mature green, MG) tomato fruit, but as fruit start their ripening program and become ripe (red ripe, RR), concurrently they become more susceptible to infections, which lead to rapid breakdown of host tissues and extensive microbial colonization (Cantu et al., 2009).

The roles of the plant stress hormones, ethylene (ET), salicylic acid (SA), jasmonic acid (JA), and abscisic acid (ABA), in the control of plant developmental processes and the initiation of defense mechanisms against necrotrophic, biotrophic, or hemibiotrophic pathogens have been documented mostly for vegetative tissues (Doares et al., 1995b; Díaz et al., 2002; Wasternack, 2007; AbuQamar et al., 2008; Asselbergh et al., 2008; Bari and Jones, 2009; Pieterse et al., 2009; Cutler et al., 2010; López-Gresa et al., 2010; El Oirdi et al., 2011; Rivas-San Vicente and Plasencia, 2011; Nambeesan et al., 2012; Pieterse et al., 2012; Vandenbussche and Van Der Straeten, 2012). However, our understanding of how these hormones influence plant–pathogen interactions in fruit is still limited.

The gaseous hormone, ET, is involved in the control of terminal developmental programs, such as organ abscission, leaf and flower senescence, and fleshy fruit ripening (Patterson and Bleecker, 2004; Barry and Giovannoni, 2007; Klee and Giovannoni, 2011; Graham et al., 2012; Pech et al., 2012; Wang et al., 2013). ET also modulates plant resistance and susceptibility to pathogens. Thus, from one point of view, ET controls a variety of immune responses in conjunction with other signaling networks; but from another perspective, it promotes senescence or ripening, processes which facilitate infection by pathogens (Van Loon et al., 2006; Cantu et al., 2009; Van Der Ent and Pieterse, 2012).

JA influences flower development and may be involved in some ripening processes, depending on the plant species (Peña-Cortés et al., 2004). The best-known function of JA is to regulate plant immune responses against insects and pathogens, particularly necrotrophs (Glazebrook, 2005; Browse, 2009). JA may also play a role in resistance against abiotic stresses, including mechanical stress, salinity, and UV irradiation (Ballaré, 2011).

SA is a phenolic compound with hormonal features that is crucial for the establishment of basal defenses, effector-triggered immunity, and both local and systemic acquired resistance (Durrant and Dong, 2004; Vlot et al., 2009). SA is typically involved in the activation of plant defenses against biotrophs and hemibiotrophs, but it also appears to enhance susceptibility to necrotrophs by antagonizing the JA signaling pathway through the regulatory protein NPR1 and by inhibition of auxin signaling (Glazebrook, 2005; Beckers and Spoel, 2006; Koornneef et al., 2008; Spoel and Dong, 2008).

ABA regulates many aspects of plant development, including seed dormancy and germination, and plays a significant role in tolerance to abiotic stress (Fujita et al., 2006; Wasilewska et al., 2008). ABA also can influence the outcome of plant–microbe interactions. Negative and positive roles have been described for this hormone depending on the pathosystem, developmental stage of the host, and/or the environmental conditions in which the plant–pathogen interaction occurs (Mauch-Mani and Mauch, 2005; Ton et al., 2009; Robert-Seilaniantz et al., 2011). In general, ABA suppresses plant resistance mechanisms by antagonizing SA- and JA/ET-dependent immune responses (Anderson et al., 2004; Mohr and Cahill, 2007; Sánchez-Vallet et al., 2012), thereby promoting susceptibility (Spoel and Dong, 2008). In addition, negative regulation involving systemic acquired resistance activation and ABA synthesis has been documented (Yasuda et al., 2008).

Genome-wide transcriptional profiling studies have been valuable in the study of hormonal signaling during plant–pathogen interactions (Glazebrook, 2005) because they enable researchers to monitor the activation or suppression of multiple pathways simultaneously. We used hybridization-based microarray data obtained from tomato fruit infected with *B. cinerea* to characterize the patterns of expression of genes involved in hormone biosynthesis and signaling to infer the potential role of stress hormones in fruit–pathogen interactions. The expression profiles of important genes were validated and extended by qRT-PCR using independent biological material at different stages of infection. We integrated the gene expression results with susceptibility phenotypes of fruit compromised in hormone synthesis and perception, in order to provide a model describing how ET, SA, JA and ABA influence the susceptibility of tomato fruit to *B. cinerea*.

## Materials and methods

### Transcriptome analysis hormone-related genes

Genes that have been previously described as involved in the synthesis, modification, signaling, and response of ET, SA, JA, and ABA were selected based on their functional annotation from the Arabidopsis Hormone Database (AHD) 2.0 (http://ahd.cbi.pku.edu.cn) (Jiang et al., 2011). The amino acid sequences of the 414 selected genes were retrieved from the Arabidopsis TAIR10 collection (http://arabidopsis.org) and used as queries in a BLASTP search (*e*-value ≤ 1e^−3^, low complexity filter “on”) against all of the predicted proteins in the tomato (*Solanum lycopersicum*) genome sequence (ITA2.3 release; http://solgenomics.net). A total of 326 sequences with identity greater than 60% and with alignment coverage more than 70% of the query length were considered putative tomato homologs of the Arabidopsis hormone-related proteins. In addition, the sequences of 19 known tomato protein gene sequences related to ET synthesis and signaling pathways were added to the dataset. Corresponding unigene sequences and Affymetrix array chip probes were then obtained, respectively, from GenBank (http://ncbi.nlm.nih.gov/genbank/) and Affymetrix (http://www.affymetrix.com/analysis/) to extract the normalized hybridization values from the microarray analysis of *Botrytis cinerea*-infected tomato fruit (Cantu et al., 2009; http://www.ncbi.nlm.nih.gov/geo/query/acc.cgi?acc=GSE14637) at the MG and RR stages and at 1 day post-inoculation (dpi). The resulting dataset (141 tomato genes) was used to identify significant (*P* ≤ 0.05) fold changes in ET, SA, JA, and ABA-related genes that are in common or uniquely regulated by infection of MG and RR fruit by *B. cinerea* and by ripening of healthy fruit.

### Plant material

The *NahG* tomato line (cv. Moneymaker) expressing the *Pseudomonas putida* SA hydroxylase gene (*NahG*) under regulation of the constitutive promoter 35S were developed by Brading et al. (2000) and kindly provided by Dr. J. Jones (John Innes Centre, Norwich, UK). The *sitiens* tomato mutant and its wild-type background cv. Moneymaker were contributed by the Tomato Genetics Research Center (TGRC; UC Davis, CA). Tomato (*Solanum lycopersicum*) cv. Ailsa Craig (AC), the *NahG* transgenic line, the *sitiens* mutant line, and their wild-type non-transgenic control line (cv. Moneymaker) were grown in greenhouse and field conditions during 2008, 2009, and 2012 in Davis, California. Fruit were tagged at 3 days post-anthesis (dpa) and harvested at 31 dpa for MG fruit and at 42 dpa for RR fruit. Ripening stages of the fruit were confirmed by the color, size, and texture.

### Fungal culture and fruit inoculation

*B. cinerea* (B05.10) was provided by Dr. J. A. L. van Kan (Department of Phytopathology, Wageningen University). Conidia, collected from sporulating cultures grown on 1% potato dextrose agar (Difco), were counted and diluted to 500 conidia μL^−1^ for inoculations. Fruit were disinfected and inoculated as in Cantu et al. (2008a). Briefly, on the day of harvest fruit were surface sterilized by submersion in a solution of 10% (v/v) bleach followed by three deionized water rinses. At the time of inoculation fruit were wounded at seven sites to a depth of 2 mm and a diameter of 1 mm. Six out of the seven sites were inoculated with 10 μL of a water suspension containing 5000 conidia of *B. cinerea* and the seventh site was mock-inoculated with 10 μL of sterile water (wounded control). Healthy fruit were not wounded or inoculated. All fruit samples were incubated at 20°C in high humidity. Susceptibility was determined daily for 3 dpi as disease incidence (percentage inoculation sites showing symptoms of tissue maceration or soft rot). The evaluation of susceptibility was repeated with three separate harvests of fruit using 10–15 fruits per experiment. The significance of the susceptibility data was analyzed by ANOVA followed by Tukey's *post-hoc* test using R (R Foundation for Statistical Computing). For percentage values, statistical analysis was carried out after angular transformation.

### Ethylene and 1-MCP treatments

Fruit were placed in air-tight chambers containing either 10 μL/L ET, low (12 nL/L), or high (450 nL/L) levels of 1-methylcyclopropene (1-MCP; SmartFresh©, kindly contributed by AgroFresh Inc.) for 18 h at 20°C. As controls, fruit at the same stage were placed in an identical closed chamber without ET or 1-MCP. Immediately after treatment, fruit were divided into three replication groups and inoculated with *B. cinerea* and assessed for disease incidence as described above.

### RNA isolation

To confirm the gene expression changes identified in the re-analysis of the microarray hybridization data, additional MG and RR fruit (cv. AC) were inoculated as above with *B. cinerea* or kept uninoculated (i.e., healthy). Fruit pericarp and epidermal tissues were collected after 1 and 3 days post-inoculation (dpi) and high-quality RNA was isolated. Five biological replicates were produced per sample and each replicate consisted of independent pools of 3–5 fruits. Two grams of tissue per sample were ground in liquid nitrogen and 10 mL of the RNA extraction buffer (CTAB 2% v/v, PVP 2% v/v, 100 mM Tris pH 8, 2 M NaCl, 25 mM EDTA, 0.5 g/L spermidine, 10 mM β-mercaptoethanol) were added. The samples were immediately incubated for 5 min at 65°C. Two extractions with one equal volume of chloroform:isoamyl alchohol (24:1, v/v) followed by centrifugation at 4000 rpm for 45 min at 4°C were performed. The supernatant was recovered and 1/10 volume of 1M KOAc was added followed by centrifugation at 4000 rpm for 20 min at 4°C. The supernatant was collected and 1/4 volume of 10 M LiCl was added. Samples were incubated overnight at −20°C and then centrifuged at 4000 rpm for 45 min at 4°C. The supernatant was discarded and the RNA pellet was further purified using the RNeasy Plant Mini Kit (Qiagen®). DNAse treatment (RNase-Free DNase Set, Qiagen®) was done in column during the purification step. The RNA was resuspended in 35 μL of nuclease-free water. The RNA concentration and purity were measured using NanoDrop 2000c Spectrophotometer (Thermo Scientific, Inc.). The RNA integrity was checked by agarose gel electrophoresis.

### Quantitative RT-PCR

cDNA was synthesized from the prepared RNA using M-MLV Reverse Transcriptase (Promega). qRT-PCR was performed on a StepOnePlus PCR System using Fast SYBR Green Master Mix (Applied Biosystems). All qRT-PCR reactions were performed with the following cycling conditions: 95°C for 10 min, followed by 40 cycles of 95°C for 3 s and 60°C for 30 s. Tomato actin (Solyc03g078400) was used as reference gene and process in parallel with the genes of interest. Primer efficiencies were calculated using 4-fold cDNA dilutions (1:1, 1:4, 1:16, 1:64, and 1:256) in duplicate as well as checking for amplification in a negative control without DNA. The efficiencies of the primer sets used in this study were all above 90% (Table S3). Specificity of the primers was checked by analyzing dissociation curves ranging from 60 to 95°C. The 2^−ΔΔ^*CT* method (Livak and Schmittgen, 2001) was used to normalize and calibrate transcript values relative to the endogenous constitutive gene (actin, Solyc03g078400) control. Within analyses, the same calibrator was used for all genes so the scales of their linearized values are comparable. Data presented is from 3 to 5 biological replicates per treatment and per stage.

## Results and discussion

### Transcriptomic analysis and validation of hormonal-related genes during fruit infection by *B. Cinerea*

Although the complete sequence of the tomato genome is available (The Tomato Genome Consortium, 2012), an integration of genome annotations with functional information is required to assign biological importance to gene sequences and generate a framework for the study of developmental processes and signaling networks. The study of stress hormonal pathways in tomato fruit has focused mainly on the characterization of ET-related genes involved in the initiation of ripening (Barry and Giovannoni, 2007; Klee and Giovannoni, 2011; Pech et al., 2012). The roles of the stress hormones, SA, JA, and ABA, for the outcomes of fruit infections have not been extensively investigated.

We previously used microarray hybridization technology to characterize the expression changes of ripening-related genes in relation to the increased susceptibility to *B. cinerea* of ripe fruit. Using RNA from tomato fruit at two ripening stages, MG and RR at 1 dpi with *B. cinerea*, we profiled the expression of several cell wall modifying genes (e.g., polygalacturonase, expansin, and glucanases) and few hormone-related genes (e.g., *ACS2*, *ACO5*, *AOS*) (Cantu et al., 2009). The shortage of functional annotations for genes represented on the microarray has limited the identification of genes involved in hormonal pathways related to stress and pathogen responses.

Here we report (1) the identification of a set of 345 hormone-related tomato genes, which includes 19 known ET-related genes and 326 tomato genes that show significant homology to Arabidopsis genes involved in ET, SA, JA, and ABA pathways; (2) the re-annotation of the hormone-related genes on the Affymetrix Tomato Chip, and (3) the transcriptional changes of these hormonal-related genes in response to *B. cinerea* using published microarray results (Cantu et al., 2009).

Hormone-related Arabidopsis gene sequences were retrieved from the AHD 2.0 (Jiang et al., 2011) and BLASTP searches were used to identify their homologous copies in the tomato genome (minimum *e*-value < 1e^−3^; alignment coverage >70% of the query length; identity >60%). We selected the AHD 2.0 because it is currently the most comprehensive and up-to-date database of hormone-related genes; it includes 1318 gene accessions for eight different plant hormones, which had been extracted from 906 scientific papers published before August 2010. From this database, we identified 128 genes related to ET, 72 genes related to SA, 55 genes related to JA, and 159 genes related to ABA pathways (Jiang et al., 2011).

**Figure 1:**
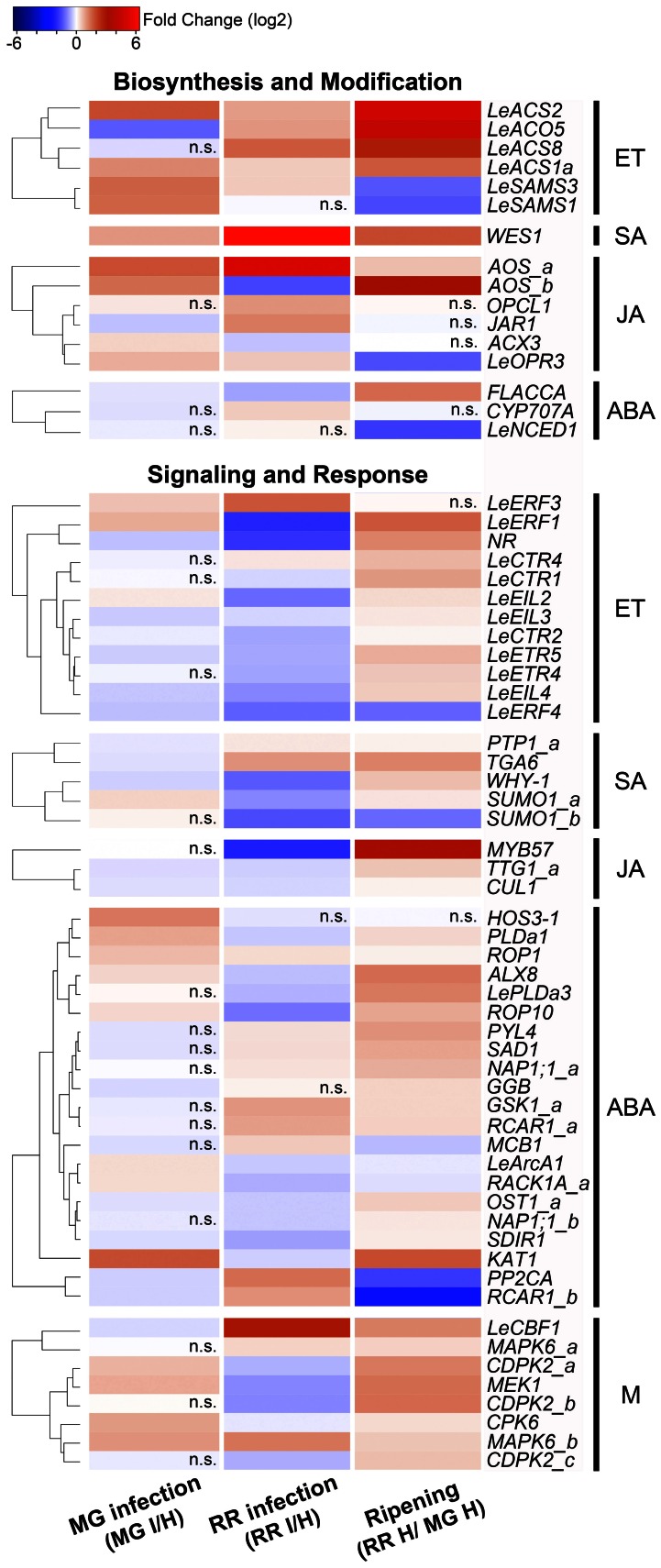
**Stress hormone-related genes identified in the microarray analysis that show expression changes as consequence of fruit infection or ripening.** Genes involved in in ethylene (ET), salicylic acid (SA), jasmonic acid (JA), abscisic acid (ABA), and multiple (M) hormonal pathways are clustered according to similarities in their expression pattern calculated by Euclidean distance. The colors in the heatmap represent the intensity of the log2-fold expression changes. Non-significant comparisons (*P* > 0.05) are marked in the figure as n.s.

Among the homologous tomato genes identified, 141 genes (Table S1) were found to be expressed in tomato fruit based on the microarray data. Of these 141 genes, we focused on those with significant changes in expression (*P* ≤ 0.05) that (1) were in common during infection of tomato fruit by *B. cinerea* regardless of the ripening stage, (2) that were responses to *B. cinerea* but are specific to the ripening stage and phenotype of the fruit (i.e., MG: resistant and RR: susceptible), and (3) that were common in response to infection and as a consequence of ripening. As result, we identified 65 stress hormone-related genes that showed differential expression in response to *B. cinerea* (Figure 1).

Relative expression changes of 20 hormone-related genes (8 ET genes, 3 SA genes, 2 JA genes, 6 ABA genes, and 1 gene related to multiple hormones) were measured by qRT-PCR using independent preparations of RNA from *B. cinerea*-infected (1 dpi) and equivalent healthy tomato fruit at MG and RR stages, in order to validate the results from the microarray analysis (Figure 3; Table S2). Additionally, gene expression was measured at 3dpi to determine whether the up- or down-regulation of the expression of these genes is maintained or modified as infection progresses (Figure 3; Table S2).

**Figure 2:**
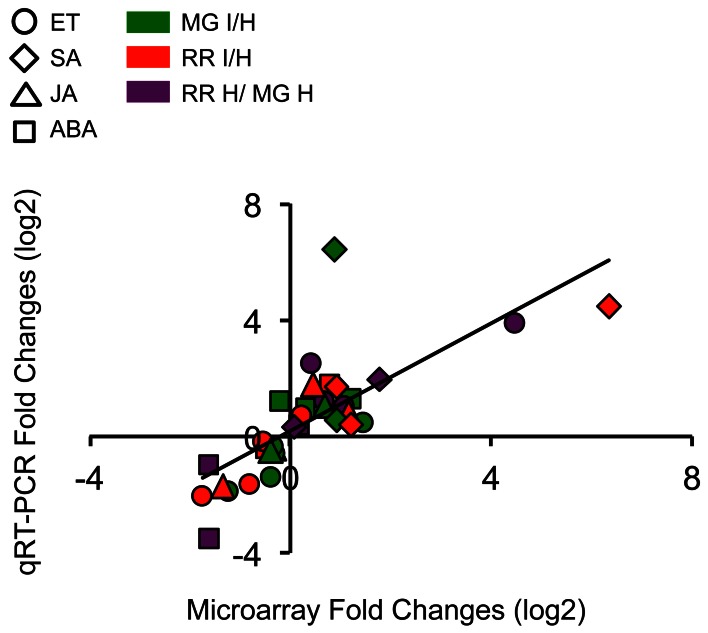
**Scatter plot shows expression changes (log2-fold) measured by microarray hybridizations and by qRT-PCR analysis of selected hormone-related genes.** Results are plotted for genes that show significant (*P* ≤ 0.05) up- or down-regulation in tomato fruit after *B. cinerea* infection and ripening. A linear trendline is shown.

For the 20 genes analyzed, 88% of all expression comparisons, i.e., infection of MG fruit (MG infected vs. healthy), infection of RR fruit (RR infected vs. healthy), and ripening (RR healthy vs. MG healthy) were observed in both the microarray and in the qRT-PCR data. However, by qRT-PCR only 59% of the gene expression changes were significant (*P* ≤ 0.05), mostly because of inter-sample variability (Table S2); in fact, the qRT-PCR coefficient of variation (CV; 20.88%) was almost three times higher than the microarray CV (7.06%). Even with the high CV of the qRT-PCR experiments, there was a strong correlation between the microarray and the qRT-PCR data (Pearson coefficient *R* = 0.76, *P* = 2.04e^−7^) (Figure 2).

In the following sections, the expression profiles of genes involved in ET, JA, SA, and ABA biosynthesis and signaling are presented and discussed in light of the susceptibility to *B. cinerea* of fruit that are either hormone-insensitive or hormone-deficient.

### Ethylene (ET)

The expression of 50% of the ET biosynthetic genes identified in fruit was altered as consequence of infection with *B. cinerea* (Figure 1; Table S1). Three patterns of transcriptional reprogramming were identified in the microarray analysis: (1) increased expression of S-adenosyl-L-methionine (SAM) synthetase genes, *LeSAMS1*, and *LeSAMS3*, which decline during ripening of healthy fruit (Van De Poel et al., 2012a); (2) up-regulation of two members of the 1-aminocyclopropane-1-carboxylic acid (ACC) synthase (ACS) gene family; and (3) down-regulation of an ACC oxidase (ACO) gene in *B. cinerea* infected MG fruit.

**Figure 3:**
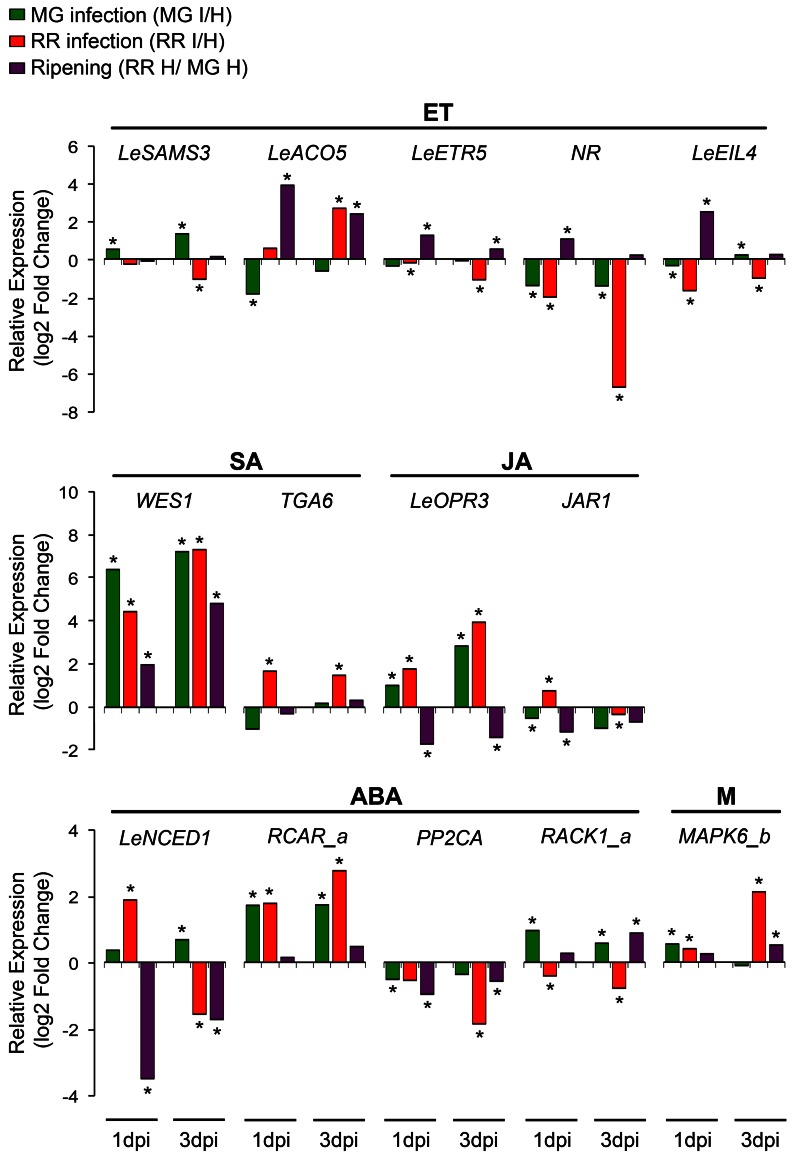
**Changes in the relative expression of representative hormone-related genes after infection of fruit by *Botrytis cinerea* and during ripening.** Changes (log2-fold) in expression of genes in ethylene (ET), salicylic acid (SA), jasmonic acid (JA), abscisic acid (ABA), and multiple (M) hormonal pathways caused by *Botrytis* infection in fruit at two ripening stages (MG I/H and RR I/H) or by ripening of healthy fruit (RR H/ MG H) were determined by qRT-PCR at two time points (1 and 3 days post-infection, dpi). Asterisks indicate significant fold changes (^*^P ≤ 0.05).

Increases in *LeSAMS1* and *LeSAMS3* expression have been detected in tomato vegetative tissues under high salinity conditions and following ABA treatment, suggesting a link between SAM and stress tolerance (Espartero et al., 1994). Besides being a substrate for ET synthesis, SAM is also utilized for the production of polyamines (PAs) and is the primary methyl-donor for modification of essential macromolecules (Van De Poel et al., 2012b). Both ET and PAs, and possibly the relative concentrations of each, mediate biotic and abiotic stress responses in fruit and vegetative tissues (Bitrián et al., 2012; Nambeesan et al., 2012). PAs have been shown to reduce the rate of fruit ripening while ET accelerates it (Mehta et al., 2002; Nambeesan et al., 2010). Therefore, enhanced SAM production and changes in the relative synthesis or abundance of ET/PA may be associated with resistance to pathogen infection, particularly in MG fruit for which the up-regulation of *LeSAMS3* after *B. cinerea* inoculation was validated by qRT-PCR; expression increased further at a later time during the infection process (i.e., 3 dpi) (Figure 3).

Tomato ACS and ACO isoforms are differentially expressed depending on the developmental process; some are specifically associated with ripening (e.g., *LeACS1a*, *LeACS2*, *LeACS4*, *LeACO1*, *LeACO3*, and *LeACO4*) while others act preferentially in vegetative tissues and immature fruit (Cara and Giovannoni, 2008; Yokotani et al., 2009; Klee and Giovannoni, 2011; Pech et al., 2012). These expression patterns relate to different systems of ET production, described later. From the microarray analysis, premature increased expression of two ACS genes involved in the tomato ripening process, *LeACS1a* and *LeACS2*, occurs in *B. cinerea*-infected MG fruit, which might suggest that pathogen infections activate the synthesis of ET, thereby accelerating the onset of the ripening process and subsequently inducing susceptibility as proposed by Cantu et al. (2009). On the other hand, down-regulation of the ET biosynthetic gene *LeACO5* only in MG fruit as consequence of infection (Figures 1, 3; Tables S1, S2) can be interpreted as a counteracting effort by the plant to control the pathogen-induced increase in ET production.

Infection of fruit affects the expression of 40% of the ET signaling components that are transcribed in fruit (Figure 1; Table S1). Expression of the ET receptors *LeETR4*, *LeETR5* and *NR* decrease after pathogen inoculation at both fruit ripening stages (Figure 1), and the down-regulation was validated in RR fruit at 1 and 3 days after *B. cinerea* infection for both *LeETR5* and *NR* genes (Figure 3; Table S2). ET receptors are negative regulators of the signaling pathway (Hua and Meyerowitz, 1998), and both their de-phosphorylation and degradation are induced upon ET binding, thereby activating responses to the hormone (Kevany et al., 2007; Kamiyoshihara et al., 2012). However, during fruit ripening, increases in the transcript levels of these receptors do not correlate with protein accumulation or receptor activity (Kevany et al., 2007). Therefore, the impact on ET perception caused by the down-regulation of the expression of the ET receptors observed during infection of fruit should be evaluated further by examining receptor protein levels and phosphorylation state. For example, the reduction in ET sensitivity caused by mutation in the *NR* receptor (i.e., constitutive receptor activation) was shown to enhance resistance of tomato leaves to several pathogens (Lund et al., 1998) and to reduce susceptibility of tomato fruit to *B*. *cinerea* infection (Cantu et al., 2009).

The expression of the primary ET response factors *LeEIL3* and *LeEIL4* is suppressed as a consequence of exposure of tomato fruit to *B. cinerea* and up-regulated during fruit ripening (Figure 1; Table S1). The down-regulation after fruit infection was validated for *LeEIL4* (Figure 3), while for *LeEIL3* only the suppression in infected MG fruit was statistically significant (Table S2). The *LeEIL1-4* genes encode redundant transcription factors that bind to secondary response elements in order to activate downstream ET responses (Tieman et al., 2001). In Arabidopsis leaves infected with the bacterial pathogen *Pseudomonas syringae*, the ET response factors EIN3 and EIL1 appear to negatively regulate plant immune responses by disrupting the pathogen-induced accumulation of SA (Chen et al., 2009). Thus, the decrease in *LeEIL4* and *LeEIL43* expression during fruit infection may represent a plant strategy to modulate the intensity of the ET response to *B. cinerea*, and/or to avoid the repression of SA biosynthesis.

The expression of other ET signaling component genes (with the exception of *LeERF4*) also is enhanced during ripening, but specific expression changes after infection depend on the ripening stage of the fruit (Figure 1; Tables S1, S2). For example, the protein kinase *LeCTR4* is up-regulated in infected RR fruit, and *LeERF1* expression increased in infected MG fruit but is reduced in infected RR fruit. Even though LeERF1 has been reported to induce fruit ripening and softening (Li et al., 2007), its over-expression also is associated with resistance of RR tomato fruit to the necrotroph, *Rhizopus nigricans* (Pan et al., 2013). In addition, ERF1 serves as an intersection point between ET and JA response pathways triggering plant defenses, particularly against necrotrophs (Lorenzo and Solano, 2005; Pieterse et al., 2012). By qRT-PCR no change in expression of *LeERF1* was detected in infected RR fruit; therefore, further analyses using additional biological material, including infections of fruit with other pathogens, are necessary to reliably assess the regulation of *ERF1* expression in responses to infections.

Experimental observations have suggested that low concentrations of ET are required to induce defense responses in fruit prior to pathogen infection (Ku et al., 1999; Akagi et al., 2011), while high and/or persistent ET levels have been related to increased pathogen susceptibility (Marcos et al., 2005). ET production in fruit is considered to be under the control of two systems, designated Systems 1 and 2. The role of each system is specific to the plant species (climacteric vs. non-climacteric) and developmental stage (Pech et al., 2012). System 1 is characterized by low levels of ET synthesis due to auto-inhibition and is present throughout early fruit development and during ripening of non-climacteric fruit (e.g., strawberry, grape, citrus, and pepper). System 2 refers to the autocatalytic synthesis of ET that is active at the onset of ripening in climacteric fruit (e.g., tomato, apple, peach, and avocado) and that leads to high levels of accumulated hormone (Yokotani et al., 2009; Klee and Giovannoni, 2011; Pech et al., 2012). It is possible that ET is generated in unripe fruit after pathogen recognition under System 1 and that this pathogen-induced concentration of ET specifically activates the expression of defense genes and/or other resistance pathways, but once the ET levels surpass a threshold, induction of System 2 and the associated climacteric ripening, or the activation of senescence/ripening pathways in non-climacteric fruit, may lead to enhanced susceptibility regardless of the defense mechanisms activated. Therefore, ET can act as a promoter of susceptibility or resistance depending on its levels in the tissue and on the developmental stage of the host; in the case of fruit, this corresponds to the point at which the tissue is competent to respond to different ET concentrations.

**Figure 4:**
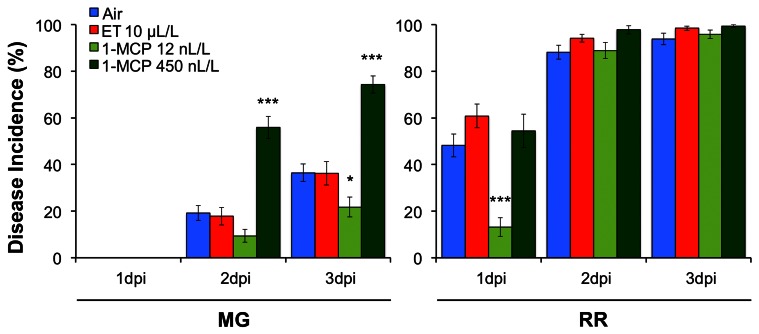
**Effect of ethylene (ET) and the ET-perception inhibitor 1-MCP on tomato fruit susceptibility to *Botrytis cinerea*.** Disease incidence (% of inoculation sites with soft rot symptoms at 1, 2, and 3 days post-inoculation, dpi) for infections of MG (31 days post-anthesis, dpa) and RR (42 dpa) wild-type tomato fruit (cv. Ailsa Craig). Immediately prior to inoculation and within 2 h of harvest, fruit were treated for 18 h with air, 10 μL/L ET and 12 nL/L 1-MCP or 450 nL/L 1-MCP. Asterisks indicate significant differences within treatments at a given time point and developmental stage (^*^P ≤ 0.05, ^***^P ≤ 0.001).

The hypothesis that ET responses during tomato fruit infection depend on the concentration and perception of this hormone is supported by the results shown in Figure 4. In this experiment, tomato fruit at MG and RR stages were pre-treated with either high levels of ET (10 μL/L), or low (12 nL/L) or high (450 nL/L) levels of the ET inhibitor, 1-MCP, prior to inoculation with *B. cinerea*. 1-MCP, which disrupts ET responses by essentially irreversibly binding to the plant cell ET receptors and maintaining their phosphorylation state (Kamiyoshihara et al., 2012), has been widely used to study ripening and disease development in fruit (Blankenship and Dole, 2003; Watkins, 2006; Cantu et al., 2009; Zhang et al., 2009b). Pre-treatment of fruit with ET had no effect on infections of MG fruit by *B. cinerea*; these fruit were about to enter the climacteric phase of ripening and were capable of perceiving the hormone. Pre-treatment with ET also did not affect infections of RR fruit, which had already established ET-induced ripening processes. Pre-treatment with low levels of 1-MCP initially reduced infections in both MG and RR fruit; however, resistance was maintained only in MG fruit in which the climacteric increase of ET was delayed. Pre-treatment with high levels of 1-MCP prematurely induced susceptibility in MG fruit but did not influence RR fruit infections. These observations suggest that low concentrations of 1-MCP may block some but not all ET receptors probably because of limited amounts of the inhibitor and continuing *de novo* generation of receptors. Thus, ET might be perceived in an appropriate concentration to promote resistance in the presence of low 1-MCP levels. In contrast, high 1-MCP levels may block ET perception longer and, thereby, hamper resistance response mechanisms that rely on ET perception. Previous studies also confirmed that application of high concentrations of 1-MCP (>450 nL/L) prior to inoculation with other pathogens (e.g., *Colletotrichum* spp., *Dothiorella* spp., *Penicillium* spp.) often induces rapid decomposition of climacteric and non-climacteric fruit, while application of low concentrations (5–100 nL/L) tends to reduce or stop infections (Ku et al., 1999; Porat et al., 1999; Hofman et al., 2001; Bower et al., 2003; Janisiewicz et al., 2003; Adkins et al., 2005; Marcos et al., 2005).

ET-mediated defenses are generally effective for controlling biotrophs, but are frequently inadequate against necrotrophs (Van Loon et al., 2006; Cantu et al., 2009; Van Der Ent and Pieterse, 2012). Certain necrotrophic pathogens, such as *Penicillium digitatum* and *B. cinerea*, are capable of producing ET, possibly as a virulence factor (Achilea et al., 1985; Cristescu et al., 2002; Zhu et al., 2012) and/or to induce ET synthesis in the host, thus promoting premature senescence or ripening (Marcos et al., 2005; Swartzberg et al., 2008; Cantu et al., 2009). However, it is not possible to distinguish experimentally in infected tissues between the ET synthesized by the pathogen or by the host. While it is known that ET is synthesized by *B. cinerea* using the 2-keto-4-methylthiobutyric acid pathway (Cristescu et al., 2002) rather than the ACC pathway used in plants, the genes responsible for ET biosynthesis by *B. cinerea* have not been identified so inferences about total ET abundance based on biosynthetic gene expression of both organisms cannot be made yet. The dissimilar roles of ET in necrotrophic and biotrophic infections may relate to the model of ET concentration-dependent responses of plant tissues. Low levels of ET may effectively control both biotrophs and necrotrophs, but higher ET levels may favor only necrotrophic infections. Whether a pathogen is capable of perceiving ET and responding to the hormone during its development or when interacting with the host is also relevant in infections and should be explored further.

### Salicylic acid (SA)

Two routes of SA biosynthesis had been described in plants, the isochorismate (IC) pathway and the phenylalanine ammonia-lyase (PAL) pathway, but neither pathway has been completely resolved (Dempsey et al., 2011). SA synthesis in response to pathogen infection and abiotic stress is apparently preferentially by the IC pathway (Wildermuth et al., 2001; Garcion et al., 2008; Tsuda et al., 2008), while the PAL pathway may have a minor contribution in local resistance (Ferrari et al., 2003). No significant changes in gene expression in either SA biosynthesis pathway were detected in the microarray analysis. Only the expression of *WES1*, a SA-modification enzyme, increased as consequence of ripening and infection, as shown in the microarray and validation studies (Figure 1; Table S1). Further up-regulation of *WES1* was also observed later in infection (3 dpi) in both MG and RR fruit (Figure 3). WES1 catalyzes SA–Asp conjugation (Zhang et al., 2007). The SA–Asp conjugate is considered to be an inactive form of SA and a target for catabolism (Dempsey et al., 2011). Thus, this result may suggest that SA inactivation occurs during fruit ripening and is a generalized response of tomato fruit to pathogen challenge regardless of the ripening stage. Moreover, SA can influence the levels of other hormones, including ET (Ding and Yi Wang, 2003), and in fruit it could interfere with the regulation of ripening. Further characterization of the SA synthesis pathways and studies of the hormone's production/modification during fruit development are needed to understand fully its impacts on fruit–pathogen interactions.

SA signaling occurs via NPR1-dependent and -independent pathways (Vlot et al., 2009). NPR1 is a transcriptional co-regulator of SA responses and has been recently identified as a receptor of SA in plants (Wu et al., 2012). In the NPR1-dependent pathway, NPR1 monomers interact with members of the TGA family of bZIP transcription factors to regulate expression of SA-responsive genes (Kesarwani et al., 2007; Vlot et al., 2009). TGA factors can be activators or repressors depending on the presence of SA and their ability to form specific protein complexes (Pontier et al., 2001; Zhang et al., 2003). From the microarray and qRT-PCR results, the down-regulation of a tomato homolog of *TGA6* in MG fruit (1 dpi) and its up-regulation in RR fruit (at 1 and 3 dpi) suggest that this gene may serve as a control point to modulate SA signaling during fruit–pathogen interactions (Figures 1, 3; Tables S1, S2). Tomato TGAs have been previously implicated in resistance against biotrophs (Ekengren et al., 2003) and can be recruited by necrotrophic pathogens to induce susceptibility (Rahman et al., 2012).

Independently from NPR1, the protein kinases MAPK3 and MAPK6 have been shown to be important in systemic acquired resistance and priming for resistance (Menke et al., 2004; Beckers et al., 2009; Galletti et al., 2011). Pre-treatment with low concentration of SA prior to pathogen encounters induces the accumulation of inactive MAPK3 and MAPK6 in vegetative tissues and once an infection occurs, these kinases are rapidly activated to enhance the expression of defense genes (Beckers et al., 2009). The phosphatases, PTP1 and MKP1, inactivate both MAPK3 and MAPK6 and therefore suppress the downstream SA signaling pathway (Bartels et al., 2009). In infected fruit, a significant decrease in expression of a *PTP1* homolog is observed only in resistant (i.e., MG) fruit, which may lead to the activation of the MAPKs. In particular, a tomato homolog of *MAPK6* (i.e., *MAPK6_b*) appears to be significantly up-regulated in MG fruit after *B. cinerea* inoculation (1 dpi) (Figures 1, 3; Tables S1, S2). These results indicate that SA responses via the MAPK pathway may be distinct from those mediated by NPR1 and that these responses may be necessary for both basal and induced defenses in MG fruit.

**Figure 5:**
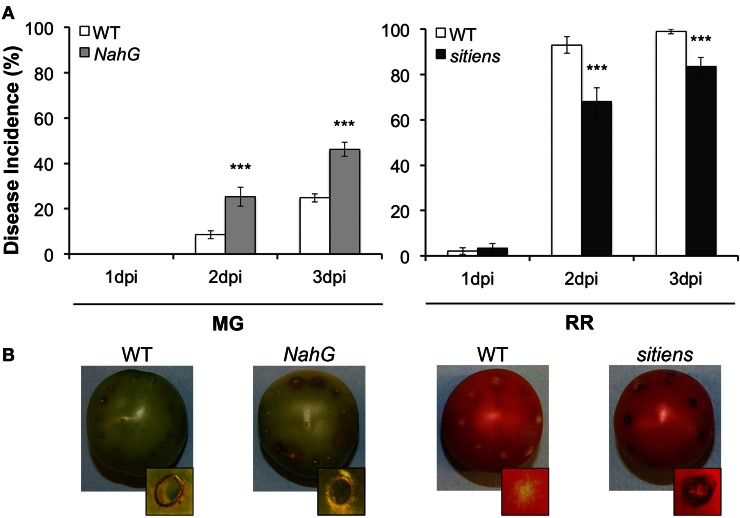
**Susceptibility of *NahG* and *sitiens* tomato fruit to *Botrytis cinerea*. (A)** Disease incidence (% of inoculation sites with soft rot symptoms at 1, 2, and 3 days post-inoculation, dpi) of *NahG* MG stage fruit (31 days post-anthesis, dpa) and *sitiens* RR stage fruit (42 dpa) compared to the isogenic wild-type (WT) cultivar Moneymaker. Asterisks indicate significant differences between genotypes at a given time point and developmental stage (^***^P ≤ 0.001). **(B)** Representative-inoculated fruit (3 dpi) for each genotype. Insets in all frames show a magnification of an inoculation site, viewed from above the fruit surface (3 dpi). WT fruit at MG stage and *sitiens* fruit at RR stage present a dark necrotic ring that limits the disease symptoms, whereas MG *NahG* fruit or RR WT fruit do not display this inoculation site-localized necrotic zone.

The susceptibility of the *NahG* tomato line, which does not accumulate SA (Brading et al., 2000), provides additional support for the hypothesis that some SA responses can contribute to resistance in fruit (Figure 5A). When we inoculated *NahG* fruit with *B. cinerea* conidia, the fruit at the MG stage were significantly more susceptible to *B. cinerea* infection than their wild-type counterparts and did not generate the localized necrotic response surrounding the inoculation site that is common in resistant unripe fruit [i.e., a lignified and suberized layer of necrotized cells; Figure 5A; (Cantu et al., 2009)]. The localized necrotic response in MG fruit is associated with an oxidative burst that is visible within 18 h after pathogen inoculation (Cantu et al., 2009), which could be potentiated by SA as part of a positive feedback loop between this hormone and reactive oxygen species (Overmyer et al., 2003; Vlot et al., 2009). On the other hand, RR fruit from *NahG* and wild-type plants were equally susceptible to *B. cinerea* and no necrotic response was evident with either genotype (data not shown). These results suggest that unripe MG fruit are capable of promoting SA-mediated responses, possibly independently from those influenced by NPR1 (e.g., MAPK-related), and thereby, may prime fruit for resistance without favoring susceptibility.

### Jasmonic acid (JA)

The increase in expression of JA biosynthetic and the subsequent accumulation of JA occurs locally as a consequence of pathogen, insect or physical damage to plant tissues (Cheong et al., 2002; Wasternack, 2007; Browse, 2009). Up-regulation of three tomato homologs encoding JA biosynthetic enzymes, allene oxide synthase (AOS), 12-oxo-cis-10,15-phytodienoic acid (OPDA) reductase 3 (OPR3), and 3-oxo-2-(cis-2'-pentenyl)-cyclopentane-1-octanoic acid (OPC)-8:CoA ligase (OPCL1) was observed during infections of MG and RR fruit (Figure 1; Table S1). The expression of the *OPR3* homolog was confirmed in *B. cinerea*-infected fruit after 1 and 3 dpi (Figure 3; Table S2). In addition, up-regulation of a *JAR1* homolog is detected in RR fruit at 1 dpi (Figures 1, 3; Tables S1, S2), but at 3 dpi its expression is down-regulated in both MG and RR tissues (Figure 3; Table S2). JAR1 is a GH3 acyl-adenylase that conjugates isoleucine to JA, activating the hormone (Staswick and Tiryaki, 2004; Thines et al., 2007) and it is required to activate JA-related responses of Arabidopsis leaves against necrotrophic infection (Staswick et al., 1998).

In the microarray data, transcriptional changes in response to *B. cinerea* are only evident for homologs of two downstream JA-responsive factors (*MYB57* and *TTG1_a*) and a member of the SCFCOI1 complex (*CUL1*). Transcriptional reprogramming of important JA-signaling components (e.g., *COI1*, *MYC2*) was not evident during tomato fruit infection or during ripening (Figure 1; Table S1), which may indicate that activation of JA-related defenses in fruit occurs *via* other signaling pathways. In contrast, when *B. cinerea* infects petunia flowers it was been reported that expression of *COI1* is activated in the absence of ET signaling (Wang et al., 2013), which indicates that JA signaling pathways could be differentially activated as consequence of fungal infection depending on the plant tissue and the presence/absence of endogenous ET levels. Both JA and ET synergistically activate the expression of a large set of defense genes (Thomma et al., 2001; Glazebrook, 2005; Lorenzo and Solano, 2005) through the transcription factors, ERF1 and ORA59 (Lorenzo and Solano, 2005; Pré et al., 2008). These shared JA- and ET-regulated responses are preferentially triggered when ET is present, while responses unique to JA are induced mostly in the absence of ET (Lorenzo et al., 2004; Pieterse et al., 2009).

SA and JA signaling pathways are generally considered antagonistic (Beckers and Spoel, 2006; Koornneef et al., 2008; Spoel and Dong, 2008; Pieterse et al., 2009, 2012). The antagonism is dependent on NPR1 and influenced by the hormone concentration and the timing of the SA/JA signal initiation (Mur et al., 2006; Koornneef et al., 2008; Leon-Reyes et al., 2010). This interplay between SA and JA might reduce fitness costs from the unnecessary deployment of defenses and could serve as a regulatory mechanism allowing plants to adjust their defense strategies in response to the pathogen's lifestyle (Pieterse et al., 2009; Van Der Ent and Pieterse, 2012). However, some pathogens can exploit the SA/JA antagonism for their own benefit (Alkan et al., 2011; El Oirdi et al., 2011); for example, *B. cinerea* produces an elicitor of SA responses through the NPR1-dependent pathway, which leads to the inactivation of two JA-response genes, *Proteinase I* and *II*, that are required for resistance against necrotrophs (El Oirdi et al., 2011).

ET can counteract the negative effects of NPR1 on JA responses, but it also enhances the NPR1-dependent expression of SA defense genes (De Vos et al., 2006; Spoel et al., 2007; Leon-Reyes et al., 2009). Leon-Reyes et al. (2010) proposed that the concurrent activation of ET and JA pathways promotes plant insensitivity to subsequent SA-mediated suppression of JA-dependent defenses, which then favors effective resistance against pathogens of different lifestyles. Hence, localized synthesis and perception of JA, ET, and SA at the appropriate relative concentration and timing appear to be required for plant resistance. During infections of fruit, ET, SA, and JA networks might interact to stimulate defenses. Nonetheless, accumulation of susceptibility factors as a consequence of ET-triggered senescence/ripening and the antagonism between SA and JA responses may represent opposing influences in the fruit–pathogen interaction and, thus, lead to susceptibility.

### Abscisic acid (ABA)

Increased expression of the tomato 9-cis-epoxycarotenoid dioxygenase 1 (*LeNCED1*), a key ABA biosynthetic gene, occurs during early infection (1 dpi) of susceptible (RR) fruit (Figures 1, 3; Tables S1, S2), which suggests a link between ABA synthesis and fruit susceptibility. Several plant pathogens, including *B. cinerea*, generate ABA during infection or use effectors to induce its production by the host, facilitating senescence/ripening and subsequent colonization of the ripened tissue (Siewers et al., 2004, 2006; De Torres-Zabala et al., 2007, 2009).

ABA has been involved in fruit ripening of climacteric and non-climacteric fruit (Zhang et al., 2009a; Koyama et al., 2010; Jia et al., 2011; Soto et al., 2013). Exogenous treatments of ABA induce the expression of the ripening-associated ET biosynthetic genes *LeACS2*, *LeACS4*, and *LeACO1*, thereby, triggering ET production and ripening (Zhang et al., 2009a). In tomato fruit, expression of the 9-cis-epoxycarotenoid dioxygenase 1 (*LeNCED1*) increases at the onset of ripening prior to the ET climacteric rise (Zhang et al., 2009a). A slight induction of *LeNCED1* was detected in infected MG fruit (1 and 3 dpi), which could have been prematurely induced to initiate climacteric ripening; however, a significant decrease in expression occurs at the late stage of ripening (Figure 3; Table S2). The development and analysis of a genetic knock-out mutant line in *LeNCED1* will be instrumental to understand the impact of ABA synthesis during the increase in ripe fruit susceptibility.

The expression of *FLACCA*, a tomato molybdenum cofactor synthase that is involved in ABA biosynthesis, increases as consequence of ripening, but it is reduced in response to the *B. cinerea* infection (Figure 1; Table S1). These observations indicate that the plant may reduce the expression of *FLACCA* in an effort to contain the rise in ABA production caused by the pathogen colonization; however, experimental evidence is needed to test this hypothesis.

The interaction between tomato fruit and *B. cinerea* results in significant changes in the expression of 37% genes involved in the ABA signaling pathway (Figure 1; Table S1). Alterations in regulators of ABA signaling/responses (e.g., receptors and transcription factors) are detected as well as changes in membrane protein channels (e.g., *KAT1*).

In general, increased expression of the *PYL/PYR/RCAR* receptors was observed in RR fruit (Figure 1; Table S1). The PYL/PYR/RCAR receptors are positive regulators of ABA response by blocking the PP2Cs inhibitors (Raghavendra et al., 2010; Cutler et al., 2010). In Arabidopsis, suppression of three PP2C clade A phosphatases results in constitutive activation of ABA signaling and increased susceptibility to fungal infection (Sánchez-Vallet et al., 2012). In agreement with these results, significant up-regulation of a *RCAR1* homolog (*RCAR_a*) and down-regulation of a PP2C homolog in infected RR fruit at 1 and 3 dpi provides further support for a positive relationship between ABA responses and susceptibility (Figure 3; Table S2).

Enhanced expression of suppressor genes (e.g., tomato homologs of *HOS3a* and *RACK1*) throughout the ABA hormone-signaling network is detected after inoculation with *B. cinerea* of resistant MG fruit (Figures 1, 3; Tables S1, S2). In contrast to the increased expression in MG fruit, the homolog *RACK1_a* is significantly down-regulated in RR fruit at 1 and 3 dpi (Figure 3; Table S2). Previous studies have demonstrated a role for RACK1 in the activation of defense mechanisms in response to pathogens in rice. The rice *RACK1* homolog (i.e., *RACK1A*) triggers ROS production, defense gene expression, and disease resistance by interacting with OsRac1, a Rac/Rop small GTPase involved in basal immune responses (Nakashima et al., 2008). It is plausible that tomato homolog of *RACK1* has a similar role in fruit by controlling infections in MG fruit.

The contribution of ABA to the enhanced susceptibility of ripe fruit is supported by the disease development assays with the tomato *sitiens* mutant which fails to synthesize ABA (Harrison et al., 2011). Inoculation of RR *sitiens* fruit with *B. cinerea* resulted in a significant decrease in disease incidence when compared to the infected wild-type RR fruit (Figure 5B). Interestingly, about 40% of the inoculated sites in RR *sitiens* fruit displayed the typical localized necrotic response of wild-type MG green fruit (Figure 5B). MG *sitiens* fruit are as resistant as MG wild-type fruit (data not shown). The molecular mechanisms that mediate the reduction of susceptibility in RR *sitiens* fruit are not known; however, analysis of necrotrophic infections in leaves of *sitiens* plants suggest that a strong induction of defense-related genes (e.g., PR-1), the oxidative burst, and an increase in cuticle permeability might be crucial for the resistant phenotype of this mutant (Asselbergh et al., 2007; Curvers et al., 2010).

## Concluding remarks

Plants modulate the ET, SA, JA, and ABA hormone networks to induce immune responses against the attacks by various classes of pathogens (Pieterse et al., 2012). Recent studies indicate that other hormones such as auxin, gibberellins, cytokinins, cell wall oligogalacturonides, and brassinosteroids might also be implicated in responses to pathogens either directly or by interacting with other hormones (Doares et al., 1995a; Bari and Jones, 2009). The interactions among hormones provide the plant with a powerful regulatory potential, but also give opportunities for pathogens to manipulate the plant defense-signaling networks to their advantage (Van Der Ent and Pieterse, 2012). Plants in their natural environments infrequently interact with a single pathogen species, rather they are impacted by microbial communities, herbivores, and other plants, all of which could individually, collectively or cooperatively influence responses to contact with pathogens. This complexity should be taken into account when studying plant–pathogen associations.

In fruit, high levels of ET and ABA, which stimulate senescence/ripening processes, may facilitate colonization by necrotrophs. The balance between SA and JA responses seems to be crucial for resistance in unripe fruit, while ABA production correlates with ripe fruit susceptibility. ET, at appropriate concentrations, also contributes to the resistance of fruit by activating JA and/or ET responses and possibly by blocking the antagonistic effect of SA on JA signaling. Hence, the role of plant hormones in promoting fruit resistance or susceptibility depends on the interaction of several factors, including: (1) the concentration of the hormones, (2) the timing of the synthesis and perception of the hormones, (3) the competence of the host tissue to respond to active forms of the hormones, (4) the localization of the plant's response to the hormones, and (5) the pathogen's infection strategy, including its own production of hormones.

**Figure 6:**
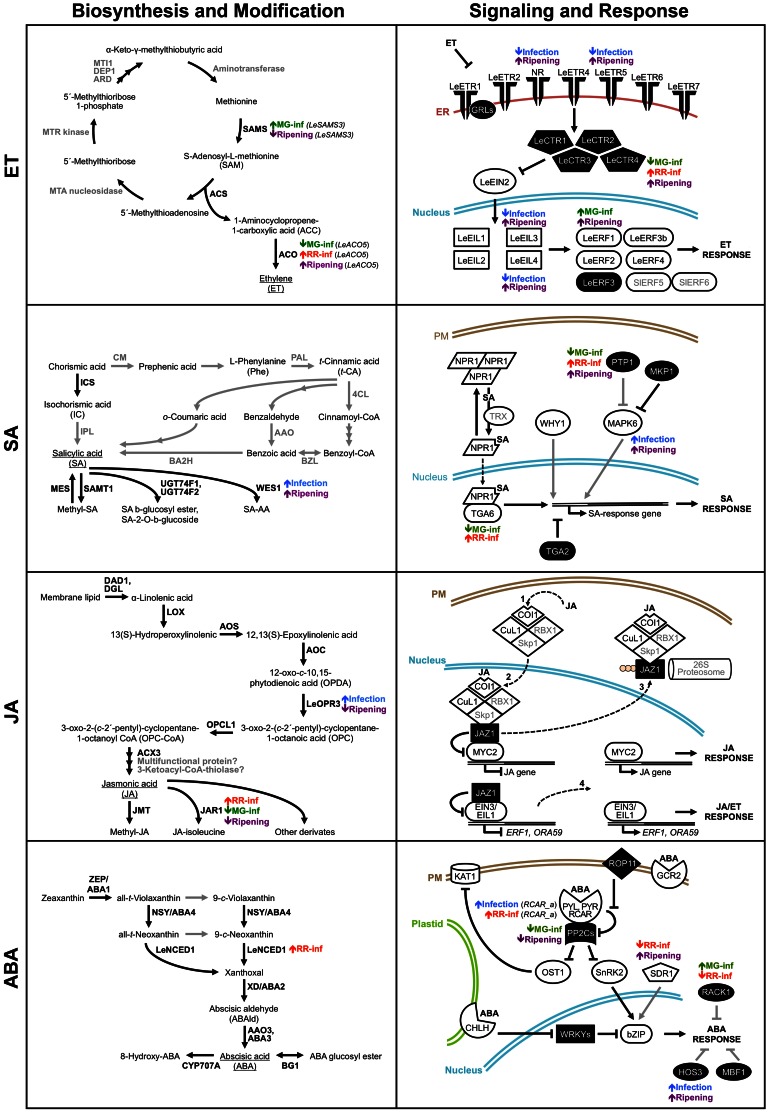
**Overview of key expression changes of genes involved in genes in ethylene (ET), salicylic acid (SA), jasmonic acid (JA), and abscisic acid (ABA) pathways during the tomato fruit–*Botrytis cinerea* interaction.** Schematic depictions of the ET, SA, JA, and ABA biosynthesis/modification and signaling/response pathways summarize the microarray analysis and qRT-PCR results and highlight changes in transcript abundance affected by fungal infection or by ripening *per se* (Cantu et al., 2009). Proteins identified in the microarray analysis with significant homology to Arabidopsis genes or known ethylene-related genes are in black bold font; whereas proteins that were not detected in our study or are hypothetical are indicated in gray bold font. Black solid lines indicate well-characterized steps or interactions, while gray solid lines refer to steps/interactions that have not been experimentally confirmed. Dashed lines refer to protein translocation between cellular compartments. In the signaling pathways, solid white figures correspond to positive regulators of hormonal responses while solid black figures indicate negative regulators. Gene expression changes caused by *B. cinerea* infections of tomato fruit at two ripening stages (MG-inf and RR-inf), that are common to infection of fruit at both stages (Infection), or that occur during ripening of healthy fruit (Ripening) are identified next to the appropriate proteins in the pathways. Up-regulation of gene expression is depicted by a short up arrow and down-regulation by a short down arrow. The detailed microarray and qRT-PCR results are presented in Tables S1, S2 in the supplementary material and the references used to build this figure are listed in Table S4.

The interaction between tomato fruit and *B. cinerea* causes transcriptional reprograming of multiple plant hormone networks simultaneously, and, depending on the developmental stage of the fruit contributes to either resistance or susceptibility outcomes. In Figure 6, we provide an overview of key expression changes of genes involved in biosynthesis, modification, signaling, and response pathways of the hormones (i.e., ET, SA, JA, and ABA) that, based on our transcriptome profiling analysis and validation, we propose to be part of the regulation of the resistance-to-susceptibility transition associated with ripening and healthy fruit ripening. Analytical methods that allow the simultaneous profiling of multiple signaling molecules that are produced during fruit infections (Müller and Munné-Bosch, 2011), will shed further light on the signaling networks that control fruit susceptibility in the context of ripening, but the challenge of identifying whether the hormones are synthesized by the host or by the pathogens will still be a limitation.

New strategies to study complex gene networks involved in hormone signaling in fruit–pathogen interactions, including the analysis of natural or induced mutants (i.e.: TILLING populations) in both plants and pathogens, the use of laser micro-dissection and cell-specific transcriptomics, and metabolomics can contribute novel important information to our understanding of the biological and ecological importance of plant development in modulating resistance and susceptibility. From an applied perspective, evaluating the specific hormonal events that promote fruit susceptibility may facilitate the development of commodities that ripen successfully and yet are less susceptible to pathogen infection.

### Conflict of interest statement

The authors declare that the research was conducted in the absence of any commercial or financial relationships that could be construed as a potential conflict of interest.

## Supplementary material

Table S1 **Microarray expression data for the 141 putative hormone-related genes expressed in fruit**. The table includes the Arabidopsis (TAIR, http://arabidopsis.org) and tomato accessions (Sol Genomics Network, http://solgenomics.net), the Affymetrix probes and annotations, the gene names, and the log2-fold changes of the comparisons between inoculated and control fruit (i.e., MG I/H and RR H/I) or during ripening (i.e., RR H/MG H). Different putative tomato homologs for the same Arabidopsis gene are distinguished by a letter after the gene name, for example *MAPK6_a* and *MAPK6_b*.

Table S2 **Candidate stress hormone-related genes used for qRT-PCR analysis**. Changes in relative expression (log2) between infected and control fruit (i.e., MG I/H and RR H/I) at 1 dpi or during ripening (i.e., RR H/MG H) of the 20 genes used in the validation of the microarray results, and their correspondent changes at 3 dpi. Non-significant changes (*P* ≤ 0.05) are indicated in gray font.

Table S3 **Primer sequences used for qRT-PCR**.

Table S4 **References utilized to build the diagrams in Figure 6**.

## References

[B1] AbuQamar S. Chai M.-F. Luo H. Song F. Mengiste T. (2008). Tomato protein kinase 1b mediates signaling of plant responses to necrotrophic fungi and insect herbivory. Plant Cell 20, 1964–1983 10.1105/tpc.108.059477 18599583 PMC2518242

[B2] Achilea O. Chalutz E. Fuchs Y. Rot I. (1985). Ethylene biosynthesis and related physiological changes in *Penicillium digitatum*-infected grapefruit (*Citrus paradisi*). Physiol. Mol. Plant Pathol. 26, 125–134

[B3] Adkins M. F. Hofman P. J. Stubbings B. A. Macnish A. J. (2005). Manipulating Avocado Fruit Ripening with 1-methylcyclopropene. Postharvest Biol. Technol. 35, 33–42

[B4] Akagi A. Dandekar A. M. Stotz H. U. (2011). Resistance of *Malus domestica* fruit to *Botrytis cinerea* depends on endogenous ethylene biosynthesis. Phytopathology 101, 1311–1321 10.1094/PHYTO-03-11-0087 21809978

[B5] Alkan N. Fluhr R. Prusky D. (2011). Ammonium secretion during *Colletotrichum coccodes* infection modulates salicylic and jasmonic acid pathways of ripe and unripe tomato fruit. Mol. Plant Microbe Interact. 25, 85–96 10.1094/MPMI-01-11-0020 22150075

[B6] Anderson J. P. Badruzsaufari E. Schenk P. M. Manners J. M. Desmond O. J. Ehlert C. (2004). Antagonistic interaction between abscisic acid and jasmonate-ethylene signaling pathways modulates defense gene expression and disease resistance in Arabidopsis. Plant Cell 16, 3460–3479 10.1105/tpc.104.025833 15548743 PMC535886

[B7] Asselbergh B. Curvers K. Franca S. C. Audenaert K. Vuylsteke M. Van Breusegem F. (2007). Resistance to *Botrytis cinerea* in *sitiens*, an abscisic acid-deficient tomato mutant, involves timely production of hydrogen peroxide and cell wall modifications in the epidermis. Plant Physiol. 144, 1863–1877 10.1104/pp.107.099226 17573540 PMC1949893

[B8] Asselbergh B. De Vleesschauwer D. Höfte M. (2008). Global Switches and Fine-Tuning ABA Modulates Plant Pathogen Defense. Mol. Plant Microbe Interact. 21, 709–719 10.1094/MPMI-21-6-0709 18624635

[B9] Ballaré C. L. (2011). Jasmonate-induced defenses: a tale of intelligence, collaborators and rascals. Trends Plant Sci. 16, 249–257 10.1016/j.tplants.2010.12.001 21216178

[B10] Bari R. Jones J. (2009). Role of plant hormones in plant defence responses. Plant Mol. Biol. 69, 473–488 10.1007/s11103-008-9435-0 19083153

[B11] Barry C. Giovannoni J. (2007). Ethylene and fruit ripening. J. Plant Growth Regul. 26, 143–159

[B12] Bartels S. Anderson J. C. González Besteiro M. A. Carreri A. Hirt H. Buchala A. (2009). MAP kinase phosphatase1 and protein tyrosine phosphatase1 are repressors of salicylic acid synthesis and SNC1-mediated responses in Arabidopsis. Plant Cell 21, 2884–2897 10.1105/tpc.109.067678 19789277 PMC2768924

[B13] Beckers G. J. M. Jaskiewicz M. Liu Y. Underwood W. R. He S. Y. Zhang S. (2009). Mitogen-activated protein kinases 3 and 6 are required for full priming of stress responses in *Arabidopsis thaliana*. Plant Cell 21, 944–953 10.1105/tpc.108.062158 19318610 PMC2671697

[B14] Beckers G. J. M. Spoel S. H. (2006). Fine-tuning plant defence signalling: salicylate versus jasmonate. Plant Biol. 8, 1–10 10.1055/s-2005-872705 16435264

[B15] Bitrián M. Zarza X. Altabella T. Tiburcio A. F. Alcázar R. (2012). Polyamines under abiotic stress: metabolic crossroads and hormonal crosstalks in plants. Metabolites 2, 516–528 10.3390/metabo2030516 PMC3901213 24957645

[B16] Blankenship S. M. Dole J. M. (2003). 1-Methylcyclopropene: a review. Postharvest Biol. Technol. 28, 1–25

[B17] Bower J. H. Biasi W. V. Mitcham E. J. (2003). Effects of ethylene and 1-MCP on the quality and storage life of strawberries. Postharvest Biol. Technol. 28, 417–423

[B18] Brading P. A. Hammond-Kosack K. E. Parr A. Jones J. D. G. (2000). Salicylic acid is not required for Cf-2- and Cf-9-dependent resistance of tomato to *Cladosporium fulvum*. Plant J. 23, 305–318 10.1046/j.1365-313x.2000.00778.x 10929124

[B19] Browse J. (2009). Jasmonate passes muster: a receptor and targets for the defense hormone. Annu. Rev. Plant Biol. 60, 183–205 10.1146/annurev.arplant.043008.092007 19025383

[B20] Cantu D. Blanco-Ulate B. Yang L. Labavitch J. M. Bennett A. B. Powell A. L. T. (2009). Ripening-regulated susceptibility of tomato fruit to *Botrytis cinerea* requires NOR but not RIN or ethylene. Plant Physiol. 150, 1434–1449 10.1104/pp.109.138701 19465579 PMC2705034

[B21] Cantu D. Vicente A. R. Greve L. C. Dewey F. M. Bennett A. B. Labavitch J. M. (2008a). The intersection between cell wall disassembly, ripening, and fruit susceptibility to *Botrytis cinerea*. Proc. Natl. Acad. Sci. U.S.A. 105, 859–864 10.1073/pnas.0709813105 18199833 PMC2242701

[B22] Cantu D. Vicente A. R. Labavitch J. M. Bennett A. B. Powell A. L. T. (2008b). Strangers in the matrix: plant cell walls and pathogen susceptibility. Trends Plant Sci. 13, 610–617 10.1016/j.tplants.2008.09.002 18824396

[B23] Cara B. Giovannoni J. J. (2008). Molecular biology of ethylene during tomato fruit development and maturation. Plant Sci. 175, 106–113

[B24] Chen H. Xue L. Chintamananid S. Germaine H. Linb H. Cuib H. (2009). ETHYLENE INSENSITIVE3 and ETHYLENE INSENSITIVE3-LIKE1 Repress SALICYLIC ACID INDUCTION DEFICIENT2 expression to negatively regulate plant innate immunity in *Arabidopsis*. Plant Cell 21, 2527–2540 10.1105/tpc.108.065193 19717619 PMC2751940

[B25] Cheong H. W. Chang H.-S. Gupta R. Wang X. Zhu T. Luan S. (2002). Transcriptional profiling reveals novel interactions between wounding, pathogen, abiotic stress, and hormonal responses in Arabidopsis. Plant Physiol. 129, 661–667 10.1104/pp.002857 12068110 PMC161692

[B26] Cristescu S. M. De Martinis D. Te Lintel Hekkert S. Parker D. H. Harren F. J. M. (2002). Ethylene production by *Botrytis cinerea in vitro* and in tomatoes. Appl. Environ. Microbiol. 68, 5342–5350 10.1128/AEM.68.11.5342-5350.2002 12406723 PMC129912

[B27] Curvers K. Seifi H. Mouille G. De Rycke R. Asselbergh B. Van Hecke A. (2010). ABA deficiency causes changes in cuticle permeability and pectin composition that influence tomato resistance to *Botrytis cinerea*. Plant Physiol. 154, 847–860 10.1104/pp.110.158972 20709830 PMC2949027

[B28] Cutler S. R. Rodriguez P. L. Finkelstein R. R. Abrams S. R. (2010). Abscisic acid: emergence of a core signaling network. Annu. Rev. Plant Biol. 61, 651–679 10.1146/annurev-arplant-042809-112122 20192755

[B29] Dalmais B. Schumacher J. Moraga J. Le Pêcheur P. Tudzynski B. Collado I. G. (2011). The *Botrytis cinerea* phytotoxin botcinic acid requires two polyketide synthases for production and has a redundant role in virulence with botrydial. Mol. Plant Pathol. 12, 564–579 10.1111/j.1364-3703.2010.00692.x 21722295 PMC6640383

[B30] De Torres-Zabala M. Bennett M. H. Truman W. H. Grant M. R. (2009). Antagonism between salicylic and abscisic acid reflects early host–pathogen conflict and moulds plant defence responses. Plant J. 59, 375–386 10.1111/j.1365-313X.2009.03875.x 19392690

[B31] De Torres-Zabala M. Truman W. Bennett M. H. Lafforgue G. Mansfield J. W. Rodriguez Egea P. (2007). *Pseudomonas syringae* pv. *tomato* hijacks the arabidopsis abscisic acid signalling pathway to cause disease. EMBO J. 26, 1434–1443 10.1038/sj.emboj.7601575 17304219 PMC1817624

[B32] De Vos M. Van Zaanen W. Koornneef A. Korzelius J. P. Dicke M. Van Loon L. C. (2006). Herbivore-induced resistance against microbial pathogens in Arabidopsis. Plant Physiol. 142, 352–363 10.1104/pp.106.083907 16829584 PMC1557608

[B33] Dempsey D. M. A. Vlot A. C. Wildermuth M. C. Klessig D. F. (2011). Salicylic acid biosynthesis and metabolism. Arabidopsis Book 9:e0156 10.1199/tab.0156 22303280 PMC3268552

[B34] Díaz J. ten Have A. van Kan J. A. L. (2002). The role of ethylene and wound signaling in resistance of tomato to *Botrytis cinerea*. Plant Physiol. 129, 1341–1351 10.1104/pp.001453 12114587 PMC166527

[B35] Ding C.-K. Yi Wang C. (2003). The dual effects of methyl salicylate on ripening and expression of ethylene biosynthetic genes in tomato fruit. Plant Sci. 164, 589–596

[B36] Doares S. H. Syrovets T. Weiler E. W. Ryan C. A. (1995a). Oligogalacturonides and chitosan activate plant defensive genes through the octadecanoid pathway. Proc. Natl. Acad. Sci. U.S.A. 92, 4095–4098 11607534 10.1073/pnas.92.10.4095 PMC41892

[B37] Doares S. H. Narvaez-Vasquez J. Conconi A. Ryan C. A. (1995b). Salicylic acid inhibits synthesis of proteinase inhibitors in tomato leaves induced by systemin and jasmonic acid. Plant Physiol. 108, 1741–1746 10.1104/pp.108.4.1741 12228577 PMC157556

[B38] Durrant W. E. Dong X. (2004). Systemic acquired resistance. Annu. Rev. Phytopathol. 42, 185–209 10.1146/annurev.phyto.42.040803.140421 15283665

[B39] Ekengren S. K. Liu Y. Schiff M. Dinesh-Kumar S. P. Martin G. B. (2003). Two MAPK cascades, NPR1, and TGA transcription factors play a role in pto-mediated disease resistance in tomato. Plant J. 36, 905–917 10.1046/j.1365-313X.2003.01944.x 14675454

[B40] El Oirdi M. El Rahman T. A. Rigano L. El Hadrami A. Rodriguez M. C. Daayf F. (2011). *Botrytis cinerea* manipulates the antagonistic effects between immune pathways to promote disease development in tomato. Plant Cell 23, 2405–2421 10.1105/tpc.111.083394 21665999 PMC3160041

[B41] Espartero J. Pintor-Toro J. A. Pardo J. M. (1994). Differential accumulation of S-adenosylmethionine synthetase transcripts in response to salt stress. Plant Mol. Biol. 25, 217–227 8018871 10.1007/BF00023239

[B42] Ferrari S. Plotnikova J. M. De Lorenzo G. Ausubel F. M. (2003). Arabidopsis local resistance to *Botrytis cinerea* involves salicylic acid and camalexin and requires EDS4 and PAD2, but not SID2, EDS5 or PAD4. Plant J. 35, 193–205 10.1046/j.1365-313X.2003.01794.x 12848825

[B43] Flors V. Leyva Mde L. Vicedo B. Finiti I. Real M. D. García-Agustín P. (2007). Absence of the Endo-β-1, 4-glucanases Cel1 and Cel2 reduces susceptibility to *Botrytis cinerea* in tomato. Plant J. 52, 1027–1040 10.1111/j.1365-313X.2007.03299.x 17916112

[B44] Fujita M. Fujita Y. Noutoshi Y. Takahashi F. Narusaka Y. Yamaguchi-Shinozaki K. (2006). Crosstalk between abiotic and biotic stress responses: a current view from the points of convergence in the stress signaling networks. Curr. Opin. Plant Biol. 9, 436–442 10.1016/j.pbi.2006.05.014 16759898

[B45] Galletti R. Ferrari S. De Lorenzo G. (2011). Arabidopsis MPK3 and MPK6 play different roles in basal and oligogalacturonide- or flagellin-induced resistance against *Botrytis cinerea*. Plant Physiol. 157, 804–814 10.1104/pp.111.174003 21803860 PMC3192574

[B46] Garcion C. Lohmann A. Lamodière E. Catinot J. Buchala A. Doermann P. (2008). Characterization and biological function of the *ISOCHORISMATE SYNTHASE2* gene of Arabidopsis. Plant Physiol. 147, 1279–1287 10.1104/pp.108.119420 18451262 PMC2442540

[B47] Giovannoni J. J. (2004). Genetic regulation of fruit development and ripening. Plant Cell 16, S170–S180 10.1105/tpc.019158 15010516 PMC2643394

[B48] Glazebrook J. (2005). Contrasting mechanisms of defense against biotrophic and necrotrophic pathogens. Annu. Rev. Phytopathol. 43, 205–227 10.1146/annurev.phyto.43.040204.135923 16078883

[B49] Govrin E. M. Rachmilevitch S. Tiwari B. S. Solomon M. Levine A. (2006). An Elicitor from *Botrytis cinerea* induces the hypersensitive response in *Arabidopsis thaliana* and other plants and promotes the gray mold disease. Phytopathology 96, 299–307 10.1094/PHYTO-96-0299 18944445

[B50] Graham L. E. Schippers J. H. M. Dijkwel P. P. Wagstaff C. (2012). Ethylene and senescence processes, in Annual Plant Reviews, ed McManus M. T. (Oxford: Wiley-Blackwell), 305–341

[B51] Harrison E. Burbidge A. Okyere J. P. Thompson A. J. Taylor I. B. (2011). Identification of the tomato ABA-deficient mutant *sitiens* as a member of the ABA-aldehyde oxidase gene family using genetic and genomic analysis. J. Plant Growth Regul. 64, 301–309

[B52] Hofman P. J. Jobin-Decor M. Meiburg G. F. Macnish A. J. Joyce D. C. (2001). Ripening and quality responses of avocado, custard apple, mango and papaya fruit to 1-methylcyclopropene. Aust. J. Exp. Agr. 41, 567–572

[B53] Hua J. Meyerowitz E. M. (1998). Ethylene responses are negatively regulated by a receptor gene family in *Arabidopsis thaliana*. Cell 94, 261–271 10.1016/S0092-8674(00)81425-7 9695954

[B54] Janisiewicz W. J. Leverentz B. Conway W. S. Saftner R. A. Reed A. N. Camp M. J. (2003). Control of bitter rot and blue mold of apples by integrating heat and antagonist treatments on 1-MCP treated fruit stored under controlled atmosphere conditions. Postharvest Biol. Technol. 29, 129–143

[B55] Jia H.-F. Chai Y.-M. Li C.-L. Lu D. Luo J.-J. Qin L. (2011). Abscisic acid plays an important role in the regulation of strawberry fruit ripening. Plant Physiol. 157, 188–199 10.1104/pp.111.177311 21734113 PMC3165869

[B56] Jiang Z. Liu X. Peng Z. Wan Y. Ji Y. He W. (2011). AHD2.0: an update version of Arabidopsis hormone database for plant systematic studies. Nucleic Acids Res. 39, D1123–D1129 10.1093/nar/gkq1066 21045062 PMC3013673

[B57] Kamiyoshihara Y. Tieman D. M. Huber D. J. Klee H. J. (2012). Ligand-induced alterations in the phosphorylation state of ethylene receptors in tomato fruit. Plant Physiol. 160, 488–497 10.1104/pp.112.202820 22797658 PMC3440222

[B58] Kesarwani M. Yoo J. Dong X. (2007). Genetic interactions of TGA transcription factors in the regulation of pathogenesis-related genes and disease resistance in Arabidopsis. Plant Physiol. 144, 336–346 10.1104/pp.106.095299 17369431 PMC1913812

[B59] Kevany B. M. Tieman D. M. Taylor M. G. Cin V. D. Klee H. J. (2007). Ethylene receptor degradation controls the timing of ripening in tomato fruit. Plant J. 51, 458–467 10.1111/j.1365-313X.2007.03170.x 17655616

[B60] Klee H. J. Giovannoni J. J. (2011). Genetics and control of tomato fruit ripening and quality attributes. Annu. Rev. Genet. 45, 41–59 10.1146/annurev-genet-110410-132507 22060040

[B61] Kleemann J. Rincon-Rivera L. J. Takahara H. Neumann U. Van Themaat E. V. L. Van Der Does H. C. (2012). Sequential delivery of host-induced virulence effectors by appressoria and intracellular hyphae of the phytopathogen *Colletotrichum higginsianum*. PLoS Pathog. 8:e1002643 10.1371/journal.ppat.1002643 22496661 PMC3320591

[B62] Koornneef A. Leon-Reyes A. Ritsema T. Verhage A. Den Otter F. C. Van Loon L. C. (2008). Kinetics of salicylate-mediated suppression of jasmonate signaling reveal a role for redox modulation. Plant Physiol. 147, 1358–1368 10.1104/pp.108.121392 18539774 PMC2442557

[B63] Koyama K. Sadamatsu K. Goto-Yamamoto N. (2010). Abscisic acid stimulated ripening and gene expression in berry skins of the cabernet sauvignon grape. Funct. Integr. Genomics 10, 367–381 10.1007/s10142-009-0145-8 19841954

[B64] Ku V. V. V. Wills R. B. H. Ben-Yehoshua S. (1999). 1-Methylcyclopropene can differentially affect the postharvest life of strawberries exposed to ethylene. HortScience 34, 119–120

[B65] Leon-Reyes A. Du Y. Koornneef A. Proietti S. Körbes A. P. Memelink J. (2010). Ethylene signaling renders the jasmonate response of Arabidopsis insensitive to future suppression by salicylic acid. Mol. Plant Microbe Interact. 23, 187–197 10.1094/MPMI-23-2-0187 20064062

[B66] Leon-Reyes A. Spoel S. H. De Lange E. S. Abe H. Kobayashi M. Tsuda S. (2009). Ethylene modulates the role of NONEXPRESSOR OF PATHOGENESIS-RELATED GENES1 in cross talk between salicylate and jasmonate signaling. Plant Physiol. 149, 1797–1809 10.1104/pp.108.133926 19176718 PMC2663751

[B67] Li Y. Zhu B. Xu W. Zhu H. Chen A. Xie Y. (2007). LeERF1 positively modulated ethylene triple response on etiolated seedling, plant development and fruit ripening and softening in tomato. Plant Cell Rep. 26, 1999–2008 10.1007/s00299-007-0394-8 17639404

[B68] Livak K. J. Schmittgen T. D. (2001). Analysis of relative gene expression data using real-time quantitative PCR and the 2-Δ Δ CT method. Methods 25, 402–408 10.1006/meth.2001.1262 11846609

[B69] López-Gresa M. P. Maltese F. Bellés J. M. Conejero V. Kim H. K. Choi Y. H. (2010). Metabolic response of tomato leaves upon different plant–pathogen interactions. Phytochem. Anal. 21, 89–94 10.1002/pca.1179 19866456

[B70] Lorenzo O. Chico J. M. Sánchez-Serrano J. J. Solano R. (2004). *JASMONATE-INSENSITIVE1* en-codes a MYC transcription factor essential to discriminate between different jasmonate-regulated defense responses in Arabidopsis. Plant Cell 16, 1938–1950 10.1105/tpc.022319 15208388 PMC514172

[B71] Lorenzo O. Solano R. (2005). Molecular players regulating the jasmonate signalling network. Curr. Opin. Plant Biol. 8, 532–540 10.1016/j.pbi.2005.07.003 16039901

[B72] Lund S. T. Stall R. E. Klee H. J. (1998). Ethylene regulates the susceptible response to pathogen infection in tomato. Plant Cell 10, 371–382 10.1105/tpc.10.3.371 9501111 PMC144005

[B73] Marcos J. F. González-Candelas L. Zacarías L. (2005). Involvement of ethylene biosynthesis and perception in the susceptibility of citrus fruits to *Penicillium digitatum* Infection and the accumulation of defence-related mRNAs. J. Exp. Bot. 56, 2183–2193 10.1093/jxb/eri218 15983011

[B74] Mauch-Mani B. Mauch F. (2005). The role of abscisic acid in plant–pathogen interactions. Curr. Opin. Plant Biol. 8, 409–414 10.1016/j.pbi.2005.05.015 15939661

[B75] Mehta R. A. Cassol T. Li N. Ali N. Handa A. K. Mattoo A. K. (2002). Engineered polyamine accumulation in tomato enhances phytonutrient content, juice quality, and vine life. Nat. Biotechnol. 20, 613–618 10.1038/nbt0602-613 12042867

[B76] Menke F. L. H. Van Pelt J. A. Pieterse C. M. J. Klessig D. F. (2004). Silencing of the mitogen-activated protein kinase *MPK6* compromises disease resistance in Arabidopsis. Plant Cell 16, 897–907 10.1105/tpc.015552 15020743 PMC412864

[B77] Mohr P. Cahill D. (2007). Suppression by ABA of salicylic acid and lignin accumulation and the expression of multiple Genes, in Arabidopsis infected with *Pseudomonas syringae* pv. tomato. Funct. Integr. Genomics 7, 181–191 10.1007/s10142-006-0041-4 17149585

[B78] Müller M. Munné-Bosch S. (2011). Rapid and sensitive hormonal profiling of complex plant samples by liquid chromatography coupled to electrospray ionization tandem mass spectrometry. Plant Methods 7:37 10.1186/1746-4811-7-37 22098763 PMC3253682

[B79] Mur L. A. J. Kenton P. Atzorn R. Miersch O. Wasternack C. (2006). The Outcomes of concentration-specific interactions between salicylate and jasmonate signaling include synergy, antagonism, and oxidative stress leading to cell death. Plant Physiol. 140, 249–262 10.1104/pp.105.072348 16377744 PMC1326048

[B80] Nakashima A. Chen L. Thao N. P. Fujiwara M. Wong H. L. Kuwano M. (2008). RACK1 functions in rice innate immunity by interacting with the Rac1 immune complex. Plant Cell 20, 2265–2279 10.1105/tpc.107.054395 18723578 PMC2553611

[B81] Nambeesan S. Abuqamar S. Laluk K. Mattoo A. K. Mickelbart M. V. Ferruzzi M. G. (2012). Polyamines attenuate ethylene-mediated defense responses to abrogate resistance to *Botrytis cinerea* in tomato. Plant Physiol. 158, 1034–1045 10.1104/pp.111.188698 22128140 PMC3271740

[B82] Nambeesan S. Datsenka T. Ferruzzi M. G. Malladi A. Mattoo A. K. Handa A. K. (2010). Overexpression of yeast spermidine synthase impacts ripening, senescence and decay symptoms in tomato. Plant J. 63, 836–847 10.1111/j.1365-313X.2010.04286.x 20584149

[B83] Overmyer K. Broschè M. Kangasjärvi J. (2003). Reactive oxygen species and hormonal control of cell death. Trends Plant Sci. 8, 335–342 10.1016/S1360-1385(03)00135-3 12878018

[B84] Pan X. Q. Fu D. Q. Zhu B. Z. Lu C. W. Luo Y.-B. (2013). Overexpression of the ethylene response factor *Slerf1* gene enhances resistance of tomato fruit to *Rhizopus nigricans*. Postharvest Biol. Technol. 75, 28–36

[B85] Patterson S. E. Bleecker A. B. (2004). Ethylene-dependent and -independent processes associated with floral organ abscission in Arabidopsis. Plant Physiol. 134, 194–203 10.1104/pp.103.028027 14701913 PMC316299

[B86] Pech J.-C. Purgatto E. Bouzayen M. Latché A. (2012). Ethylene and fruit ripening, in Annual Plant Reviews, ed McManus M. T. (Oxford: Wiley-Blackwell), 275–304

[B87] Peña-Cortés H. Barrios P. Dorta F. Polanco V. Sánchez C. Sánchez E. (2004). Involvement of jasmonic acid and derivatives in plant response to pathogen and insects and in fruit ripening. J. Plant Growth Regul. 23, 246–260

[B88] Perfect S. E. Hughes H. B. O'Connell R. J. Green J. R. (1999). *Colletotrichum*: a model genus for studies on pathology and fungal–plant interactions. Fungal Genet. Biol. 27, 186–198 10.1006/fgbi.1999.1143 10441444

[B89] Pieterse C. M. J. Leon-Reyes A. Van Der Ent S. Van Wees S. C. M. (2009). Networking by small-molecule hormones in plant immunity. Nat. Chem. Biol. 5, 308–316 10.1038/nchembio.164 19377457

[B90] Pieterse C. M. J. Van der Does D. Zamioudis C. Leon-Reyes A. Van Wees S. C. M. (2012). Hormonal modulation of plant immunity. Annu. Rev. Cell Dev. Biol. 28, 489–521 10.1146/annurev-cellbio-092910-154055 22559264

[B91] Pontier D. Miao Z.-H. Lam E. (2001). Trans-dominant suppression of plant TGA factors reveals their negative and positive roles in plant defense responses. Plant J. 27, 529–538 10.1046/j.1365-313X.2001.01086.x 11576436

[B92] Porat R. Weiss B. Cohen L. Daus A. Goren R. Droby S. (1999). Effects of ethylene and 1-Methylcyclopropene on the postharvest qualities of ‘Shamouti’ oranges. Postharvest Biol. Technol. 15, 155–163

[B93] Powell A. L. T. Van Kan J. A. L. Ten Have A. Visser J. Greve L. C. Bennett A. B. (2000). Transgenic expression of pear *PGIP* in tomato limits fungal colonization. Mol. Plant Microbe Interact. 13, 942–950 10.1094/MPMI.2000.13.9.942 10975651

[B94] Pré M. Atallah M. Champion A. De Vos M. Pieterse C. M. J. Memelink J. (2008). The AP2/ERF domain transcription factor ORA59 integrates jasmonic acid and ethylene signals in plant defense. Plant Physiol. 147, 1347–1357 10.1104/pp.108.117523 18467450 PMC2442530

[B95] Prusky D. Lichter A. (2007). Activation of quiescent infections by postharvest pathogens during transition from the biotrophic to the necrotrophic stage. FEMS Microbiol. Lett. 268, 1–8 10.1111/j.1574-6968.2006.00603.x 17227463

[B96] Raghavendra A. S. Gonugunta V. K. Christmann A. Grill E. (2010). ABA perception and signalling. Trends Plant Sci. 15, 395–401 10.1016/j.tplants.2010.04.006 20493758

[B97] Rahman T. A. E. Oirdi M. E. Gonzalez-Lamothe R. Bouarab K. (2012). Necrotrophic pathogens use the salicylic acid signaling pathway to promote disease development in tomato. Mol. Plant Microbe Interact. 25, 1584–1593 10.1094/MPMI-07-12-0187-R 22950753

[B98] Rivas-San Vicente M. Plasencia J. (2011). Salicylic acid beyond defence: its role in plant growth and development. J. Exp. Bot. 62, 3321–3338 10.1093/jxb/err031 21357767

[B99] Robert-Seilaniantz A. Grant M. Jones J. D. G. (2011). Hormone crosstalk in plant disease and defense: more than just jasmonate-salycilate antagonism. Annu. Rev. Phytopathol. 49, 317–343 10.1146/annurev-phyto-073009-114447 21663438

[B100] Sánchez-Vallet A. López G. Ramos B. Delgado-Cerezo M. Riviere M.-P. Llorente F. (2012). Disruption of abscisic acid signalling constitutively activates arabidopsis resistance to the necrotrophic fungus *Plectosphaerella cucumerina*. Plant Physiol. 160, 2109–2124 10.1104/pp.112.200154 23037505 PMC3510135

[B101] Siewers V. Kokkelink L. Smedsgaard J. Tudzynski P. (2006). Identification of an abscisic acid gene cluster in the grey mold *Botrytis cinerea*. Appl. Environ. Microbiol. 72, 4619–4626 10.1128/AEM.02919-05 16820452 PMC1489360

[B102] Siewers V. Smedsgaard J. Tudzynski P. (2004). The P450 monooxygenase *Bc*ABA1 Is essential for abscisic acid biosynthesis in *Botrytis cinerea*. Appl. Environ. Microbiol. 70, 3868–3876 10.1128/AEM.70.7.3868-3876.2004 15240257 PMC444755

[B103] Soto A. Ruiz K. B. Ravaglia D. Costa G. Torrigiani P. (2013). ABA may promote or delay peach fruit ripening through modulation of ripening- and hormone-related gene expression depending on the developmental stage. Plant Physiol. Biochem. 64, 11–24 10.1016/j.plaphy.2012.12.011 23337357

[B104] Spoel S. H. Dong X. (2008). Making sense of hormone crosstalk during plant immune responses. Cell Host Microbe 3, 348–351 10.1016/j.chom.2008.05.009 18541211

[B105] Spoel S. H. Johnson J. S. Dong X. (2007). Regulation of tradeoffs between plant defenses against pathogens with different lifestyles. Proc. Natl. Acad. Sci. U.S.A. 104, 18842–18847 10.1073/pnas.0708139104 17998535 PMC2141864

[B106] Staswick P. E. Tiryaki I. (2004). The oxylipin signal jasmonic acid is activated by an enzyme that conjugates it to isoleucine in Arabidopsis. Plant Cell 16, 2117–2127 10.1105/tpc.104.023549 15258265 PMC519202

[B107] Staswick P. E. Yuen G. Y. Lehman C. C. (1998). Jasmonate signaling mutants of *Arabidopsis* are susceptible to the soil fungus *Pythium irregulare*. Plant J. 15, 747–754 10.1046/j.1365-313X.1998.00265.x 9807813

[B108] Swartzberg D. Kirshner B. Rav-David D. Elad Y. Granot D. (2008). *Botrytis cinerea* induces senescence and is inhibited by autoregulated expression of the *IPT* gene. Eur. J. Plant Pathol. 120, 289–297

[B108a] The Tomato Genome Consortium. (2012). The tomato genome sequence provides insights into fleshy fruit evolution. Nature 485, 635–641 10.1038/nature11119 22660326 PMC3378239

[B109] Thines B. Katsir L. Melotto M. Niu Y. Mandaokar A. Liu G. (2007). JAZ repressor proteins are targets of the SCFCOI1 complex during jasmonate signalling. Nature 448, 661–665 10.1038/nature05960 17637677

[B110] Thomma B. P. Penninckx I. A. Cammue B. P. Broekaert W. F. (2001). The complexity of disease signaling in Arabidopsis. Curr. Opin. Immunol. 13, 63–68 10.1016/S0952-7915(00)00183-7 11154919

[B111] Tieman D. M. Ciardi J. A. Taylor M. G. Klee H. J. (2001). Members of the tomato *LeEIL* (*EIN3-like*) gene family are functionally redundant and regulate ethylene responses throughout plant development. Plant J. 26, 47–58 10.1046/j.1365-313x.2001.01006.x 11359609

[B112] Ton J. Flors V. Mauch-Mani B. (2009). The multifaceted role of ABA in disease resistance. Trends Plant Sci. 14, 310–317 10.1016/j.tplants.2009.03.006 19443266

[B113] Tsuda K. Sato M. Glazebrook J. Cohen J. D. Katagiri F. (2008). Interplay between MAMP-triggered and SA-mediated defense responses. Plant J. 53, 763–775 10.1111/j.1365-313X.2007.03369.x 18005228

[B114] Vandenbussche F. Van Der Straeten D. (2012). The Role of Ethylene in Plant Growth and Development, in Annual Plant Reviews, ed McManus M. T. (Oxford: Wiley-Blackwell), 219–242

[B115] Van De Poel B. Bulens I. Markoula A. Hertog M. L. Dreesen R. Wirtz M. (2012a). Targeted systems biology profiling of tomato fruit reveals coordination of the yang cycle and a distinct regulation of ethylene biosynthesis during postclimacteric ripening. Plant Physiol. 160, 1498–1514 10.1104/pp.112.206086 22977280 PMC3490579

[B116] Van De Poel B. Bulens I. Oppermann Y. Hertog M. L. Nicolai B. M. Sauter M. (2012b). S-adenosyl-l-methionine usage during climacteric ripening of tomato in relation to ethylene and polyamine biosynthesis and transmethylation capacity. Physiol. Plant. [Epub ahead of print]. 10.1111/j.1399-3054.2012.01703.x 23020643

[B117] Van Der Ent S. Pieterse C. M. J. (2012). Ethylene: multi-tasker in plant–attacker interactions, in Annual Plant Reviews, ed McManus M. T. (Oxford: Wiley-Blackwell), 343–377

[B118] Van Kan J. A. L. (2006). Licensed to kill: the lifestyle of a necrotrophic plant pathogen. Trends Plant Sci. 11, 247–253 10.1016/j.tplants.2006.03.005 16616579

[B119] Van Loon L. C. Geraats B. P. J. Linthorst H. J. M. (2006). Ethylene as a modulator of disease resistance in plants. Trends Plant Sci. 11, 184–191 10.1016/j.tplants.2006.02.005 16531096

[B120] Vlot A. C. Dempsey D. M. A. Klessig D. F. (2009). Salicylic Acid, a multifaceted hormone to combat disease. Annu. Rev. Phytopathol. 47, 177–206 10.1146/annurev.phyto.050908.135202 19400653

[B121] Wang H. Liu G. Li C. Powell A. L. T. Reid M. S. Zhang Z. (2013). Defence responses regulated by jasmonate and delayed senescence caused by ethylene receptor mutation contribute to the tolerance of petunia to *Botrytis cinerea*. Mol. Plant Pathol. 14, 453–469 10.1111/mpp.12017 23437935 PMC6638649

[B122] Wasilewska A. Vlad F. Sirichandra C. Redko Y. Jammes F. Valon C. (2008). An update on abscisic acid signaling in plants and more …. Mol. Plant 1, 198–217 10.1093/mp/ssm022 19825533

[B123] Wasternack C. (2007). Jasmonates: an update on biosynthesis, signal transduction and action in plant stress response, growth and development. Ann. Bot. 100, 681–697 10.1093/aob/mcm079 17513307 PMC2749622

[B124] Watkins C. B. (2006). The use of 1-methylcyclopropene (1-MCP) on fruits and vegetables. Biotechnol. Adv. 24, 389–409 10.1016/j.biotechadv.2006.01.005 16530376

[B125] Wildermuth M. C. Dewdney J. Wu G. Ausubel F. M. (2001). Isochorismate synthase is required to synthesize salicylic acid for plant defence. Nature 414, 562–565 10.1038/35107108 11734859

[B126] Wu Y. Zhang D. Chu J. Y. Boyle P. Wang Y. Brindle I. D. (2012). The Arabidopsis NPR1 protein is a receptor for the plant defense hormone salicylic acid. Cell Rep. 1, 639–647 10.1016/j.celrep.2012.05.008 22813739

[B127] Yasuda M. Ishikawa A. Jikumaru Y. Seki M. Umezawa T. Asami T. (2008). antagonistic interaction between systemic acquired resistance and the abscisic acid–mediated abiotic stress response in Arabidopsis. Plant Cell 20, 1678–1692 10.1105/tpc.107.054296 18586869 PMC2483369

[B128] Yokotani N. Nakano R. Imanishi S. Nagata M. Inaba A. Kubo Y. (2009). Ripening-associated ethylene biosynthesis in tomato fruit is autocatalytically and developmentally regulated. J. Exp. Bot. 60, 3433–3442 10.1093/jxb/erp185 19605457 PMC2724697

[B129] Zhang M. Yuan B. Leng P. (2009a). Cloning of *9-cis-epoxycarotenoid dioxygenase* (*NCED*) gene and the role of ABA on fruit ripening. Plant Signal Behav. 4, 460–463 10.1093/jxb/erp026 19816120 PMC2676767

[B130] Zhang Z. Huber D. J. Hurr B. M. Rao J. (2009b). Delay of tomato fruit ripening in response to 1-methylcyclopropene is influenced by internal ethylene levels. Postharvest Biol. Technol. 54, 1–8

[B131] Zhang Y. Tessaro M. J. Lassner M. Li X. (2003). Knockout analysis of arabidopsis transcription factors *TGA2, TGA5*, and *TGA6* reveals their redundant and essential roles in systemic acquired resistance. Plant Cell 15, 2647–2653 10.1105/tpc.014894 14576289 PMC280568

[B132] Zhang Z. Li Q. Li Z. Staswick P. E. Wang M. Zhu Y. (2007). Dual regulation role of GH3.5 in salicylic acid and auxin signaling during Arabidopsis-*Pseudomonas syringae* interaction. Plant Physiol. 145, 450–464 10.1104/pp.107.106021 17704230 PMC2048736

[B133] Zhu P. Xu L. Zhang C. Toyoda H. Gan S.-S. (2012). Ethylene produced by *Botrytis cinerea* can affect early fungal development and can be used as a marker for infection during storage of grapes. Postharvest Biol. Technol. 66, 23–29

