# Supplementary material for: Tomato transcriptome and mutant analyses suggest a role for plant stress hormones in the interaction between fruit and Botrytis cinerea
Source: Front Plant Sci. 2013 May 14;4:142. doi: 10.3389/fpls.2013.00142 (PMC3653111; doi:10.3389/fpls.2013.00142)
Supplement: Table S3 — Primer sequences used for qRT-PCR. [file DataSheet3.PDF]

| Hormone | Pathway | Tomato Accesion | Gene Name | Primer Sequences (5'-3') Primer F | Primer R | Reference |
|---|---|---|---|---|---|---|
| ET | Biosynthesis/Modification | Solyc07g026650 | *LeACO5* | ACTACGAAGCCAACATGAAGAAG | GACGATGCCAAACAAAGAATG | Van de Poel et al. 2013 |
| ET | Biosynthesis/Modification | Solyc09g008280 | *LeSAMS3* | ATTGGTGCTGGAAGAAGACAAC | GCAGAAAAGGGTGAAGAAAGAA | Van de Poel et al. 2013 |
| ET | Signaling/Response | Solyc10g085570 | *LeCTR4* | GAAAAGGGGGAAGTTGGGGAA | GTTGCCGGAGATGGAGGAAA | |
| ET | Signaling/Response | AF328786.1 | *LeEIL3* | CCTCAGCAACAAACAGTTGAACTT | AACAGGCATGCTGGTTTCTTG | Tieman et al. 2001 |
| ET | Signaling/Response | Solyc06g073730 | *LeEIL4* | GACACCACACCGAAGCAAGA | TGCCAATCTCGGGGAACAAG | |
| ET | Signaling/Response | Solyc11g006180 | *LeETR5* | AGTCATCTTTTAGGAAACGCATGTT | AGGAGTACATGAAGGCCTCTGAA | Kevany et al. 2007 |
| ET | Signaling/Response | Solyc09g075440 | *NR* | AGGGAACCACTGTCACGTTTG | CTCTGGGAGGCATAGGTAGCA | Kevany et al. 2007 |
| ET | Signaling/Response | Solyc03g093610 | *LeERF1* | AAGTGGCTCGCCTAAGAGGA | TAACATTTGGTCCCCGGCTC | |
| SA | Biosynthesis/Modification | Solyc02g064830 | *WES1* | GGTTTCACAACTCTGCCCCT | GGAATGGTTTTGGTGTCGGC | |
| SA | Signaling/Response | Solyc06g065480 | *PTP1a* | TGCGATGTCTGGAGGTGAAC | TCACGAACAGCAAGAGTGTCC | |
| SA | Signaling/Response | Solyc10g080770 | *TGA6* | ATCACATGGGCACAGAAGGG | CGTCAAATCCCACTGCTGGA | |
| JA | Biosynthesis/Modification | Solyc10g009600 | *JAR1* | AATGTAGATGGCGTGGAGGC | CGGGAGTGCGATTGTGGAAT | |
| JA | Biosynthesis/Modification | Solyc07g007870 | *LeOPR3* | TGGGTTTCCTCATGTGCCAG | GCAGCTCCAGCAGGTTGATA | |
| ABA | Biosynthesis/Modification | Solyc07g056570 | *LeNCED1* | TGGGCTCTTCGGACTTGTTG | TTTAAGATCGCCGGTGGGTG | |
| ABA | Signaling/Response | Solyc03g044910 | *HOS3* | TCGTCCTCTTCCTCTGGGTC | GGTATCACGGATCTCTGCCG | |
| ABA | Signaling/Response | Solyc05g052980 | *PP2Ca* | TCGTGCCGTCCTCTGTAGAA | CGGCCACCAGCTTCTTGTAT | |
| ABA | Signaling/Response | Solyc03g119040 | *RACK1A_a* | TGTATCGTCGTCTCACTTGCC | ATCGGTGTGGGCTTTCATGG | |
| ABA | Signaling/Response | Solyc03g007310 | *RCAR1_a* | GCTGTGCAAGACAGGACAGA | ATACAGTCGGCCTCCAAAGC | |
| ABA | Signaling/Response | Solyc05g053040 | *SDIR1* | GGTGGCCTTCTTGGTCCTAC | AGAATCCAGTGCCCTCAACG | |
| Multiple | Signaling/Response | Solyc11g072630 | *MAPK6_b* | GGTTCACCGGAGGAGTCTGA | GCTTTGGAACTTGAGGCAGC | |
