# Supplementary material for: Tomato transcriptome and mutant analyses suggest a role for plant stress hormones in the interaction between fruit and Botrytis cinerea
Source: Front Plant Sci. 2013 May 14;4:142. doi: 10.3389/fpls.2013.00142 (PMC3653111; doi:10.3389/fpls.2013.00142)
Supplement: Table S4 — References utilized to build the diagrams in Figure 6. [file DataSheet4.PDF]

	Biosynthesis and Modification Pathways	Signaling Pathways
**Ethylene**	Crozier, A., Y. Kamiya, G. Bishop, and T. Yokota. (2000). "Biosynthesis of Hormones and Elicitor Molecules" in *Biochemistry and Molecular Biology of Plants,* eds. B.B Buchanan, W. Gruissem, and R.L. Jones. Rockville, MD: American Society of Plant Physiologists, 850-929.<br>Pommerrenig, B., Feussner, K., Zierer, W., Rabinovych, V., Klebl, F., Feussner, I., and Sauer, N. (2011). Phloem-specific Expression of Yang Cycle Genes and Identification of Novel Yang Cycle Enzymes in *Plantago* and *Arabidopsis*. *Plant Cell*, *23*, 1904-1919.	Cara, B., and Giovannoni, J. J. (2008). Molecular Biology of Ethylene during Tomato Fruit Development and Maturation. *Plant Science*, *175*, 106-113.<br>Lee, J. M., Joung, J. G., McQuinn, R., Chung, M. Y., Fei, Z., Tieman, D., Klee H., and Giovannoni, J. (2012). Combined Transcriptome, Genetic Diversity and Metabolite Profiling in Tomato Fruit Reveals that the Ethylene Response Factor SlERF6 Plays an Important Role in Ripening and Carotenoid Accumulation. *Plant J*., 70, 191-204.<br>Ma, Q., Du, W., Brandizzi, F., Giovannoni, J. J., and Barry, C. S. (2012). Differential Control of Ethylene Responses by GREEN-RIPE and GREEN-RIPE LIKE1 Provides Evidence for Distinct Ethylene Signaling Modules in Tomato. *Plant Physiol.*, *160*, 1968-1984.<br>Pan, Y., Seymour, G. B., Lu, C., Hu, Z., Chen, X., and Chen, G. (2012). An Ethylene Response Factor (ERF5) Promoting Adaptation to Drought and Salt Tolerance in Tomato. *Plant Cell Rep.*, 1-12.<br>Qiao, H., Shen, Z., Huang, S. S. C., Schmitz, R. J., Urich, M. A., Briggs, S. P., and Ecker, J. R. (2012). Processing and Subcellular Trafficking of ER-Tethered EIN2 Control Response to Ethylene Gas. *Science*, 19, 390-393.<br>Wang, K. L. C., Li, H., and Ecker, J. R. (2002). Ethylene Biosynthesis and Signaling Networks. *Plant Cell*, 14, S131-S151.<br>Yoo, S. D., Cho, Y., and Sheen, J. (2009). Emerging Connections in the Ethylene Signaling Network. *Trends Plant Sci.*, *14*, 270-279.

	Biosynthesis and Modification Pathways	Signaling Pathways
**SA**	Dempsey, D.M.A., Vlot, A.C., Wildermuth, M.C., and Klessig, D.F. (2011). Salicylic Acid Biosynthesis and Metabolism. *Arabidopsis Book* 9, e0156.<br>Vlot, A.C., Dempsey, D.M.A., and Klessig, D.F. (2009). Salicylic Acid, a Multifaceted Hormone to Combat Disease. *Annu. Rev. Phytopathol.* 47**,** 177-206.	Astier, J., Kulik, A., Koen, E., Besson-Bard, A., Bourque, S., Jeandroz, S., Lamotte, O., and Wendehenne, D. (2012). Protein S-nitrosylation: What's Going on in Plants? *Free Radical Bio Med. 53, 1101-1110.*<br>Bartels, S., Anderson, J.C., González Besteiro, M.A., Carreri, A., Hirt, H., Buchala, A., Métraux, J.-P., Peck, S.C., and Ulm, R. (2009). MAP KINASE PHOSPHATASE1 and PROTEIN TYROSINE PHOSPHATASE1 Are Repressors of Salicylic Acid Synthesis and SNC1-Mediated Responses in Arabidopsis. *Plant Cell* 21**,** 2884-2897.<br>Desveaux, D., Subramaniam, R., Desprès, C., Mess, J.-N., Lèvesque, C., Fobert, P.R., Dangl, J.L., and Brisson, N. (2004). A Whirly Transcription Factor is Required for Salicylic Acid-Dependent Disease Resistance in Arabidopsis. *Dev. Cell* 6**,** 229-240.<br>Galletti, R., Ferrari, S., and De Lorenzo, G. (2011). Arabidopsis MPK3 and MPK6 Play Different Roles in Basal and Oligogalacturonide- or Flagellin-Induced Resistance against *Botrytis cinerea*. *Plant Physiol.* 157**,** 804-814.<br>Menke, F.L.H., Van Pelt, J.A., Pieterse, C.M.J., and Klessig, D.F. (2004). Silencing of the Mitogen-Activated Protein Kinase *MPK6* Compromises Disease Resistance in Arabidopsis. *Plant Cell* 16**,** 897-907.<br>Rochon, A., Boyle, P., Wignes, T., Fobert, P. R., and Després, C. (2006). The Coactivator Function of Arabidopsis NPR1 Requires the Core of its BTB/POZ Domain and the Oxidation of C-terminal Cysteines. *Plant Cell*, *18*, 3670-3685.<br>Spoel, S. H., Koornneef, A., Claessens, S. M., Korzelius, J. P., Van Pelt, J. A., Mueller, M. J., Buchala, A.J., Métraux, J.P., Brown R., Kazan, K., Van Loon, L.C., Dong, X., and Pieterse, C. M. (2003). NPR1 Modulates Cross-Talk between Salicylate- and Jasmonate-Dependent Defense Pathways through a Novel Function in the Cytosol. *Plant Cell*, *15*, 760-770.<br>Tada, Y., Spoel, S. H., Pajerowska-Mukhtar, K., Mou, Z., Song, J., Wang, C., Zuo, X., and Dong, X. (2008). Plant immunity requires conformational charges of NPR1 via S-nitrosylation and thioredoxins. *Science Signal. 321*, 952-956.<br>Vlot, A.C., Dempsey, D.M.A., and Klessig, D.F. (2009). Salicylic Acid, a Multifaceted Hormone to Combat Disease. *Annu. Rev. Phytopathol.* 47**,** 177-206.<br>Wu, Y., Zhang, D., Chu, J. Y., Boyle, P., Wang, Y., Brindle, I. D., De Luca, V., and Després, C. (2012). The Arabidopsis NPR1 Protein Is a Receptor for the Plant Defense Hormone Salicylic Acid. *Cell Rep*. 1, 639-647.

	Biosynthesis and Modification Pathways	Signaling Pathways
**JA**	Crozier, A., Y. Kamiya, G. Bishop, and T. Yokota. (2000). "Biosynthesis of Hormones and Elicitor Molecules" in *Biochemistry and Molecular Biology of Plants,* eds. B.B Buchanan, W. Gruissem, and R.L. Jones. Rockville, MD: American Society of Plant Physiologists, 850-929.<br>Kombrink, E. (2012). Chemical and Genetic Exploration of Jasmonate Biosynthesis and Signaling Paths. *Planta*, 236, 1351-1366.	Devoto, A., and Turner, J. G. (2003). Regulation of Jasmonate-mediated Plant Responses in Arabidopsis. *Annals of Botany*, *92*, 329-337.<br>Kazan, K., and Manners, J. M. (2008). Jasmonate Signaling: Toward an Integrated View. *Plant Physiol.* 146, 1459-1468.<br>Katsir, L., Chung, H. S., Koo, A. J., and Howe, G. A. (2008). Jasmonate Signaling: A Conserved Mechanism of Hormone Sensing. *Curr. Opin. Plant Biol. 11*, 428-435.<br>Lorenzo, O., and Solano, R. (2005). Molecular Players Regulating the Jasmonate Signalling Network. *Curr. Opin. Plant Biol.* 8, 532-540.<br>Pauwels, L., Inzé, D., and Goossens, A. (2009). Jasmonate-inducible gene: what does it mean?. *Trends Plant Sci. 14*, 87-91.<br>Pieterse, C.M.J., Leon-Reyes, A., Van Der Ent, S., and Van Wees, S.C.M. (2009). Networking by Small-Molecule Hormones in Plant Immunity. *Nat. Chem. Biol.* 5, 308-316.<br>Thines, B., Katsir, L., Melotto, M., Niu, Y., Mandaokar, A., Liu, G., Nomura, K., He, S.Y., Howe, G. A., and Browse, J. (2007). JAZ Repressor Proteins are Targets of the SCFCOI1 Complex during Jasmonate Signalling. *Nature*, 448, 661-665.

	Biosynthesis and Modification Pathways	Signaling Pathways
**ABA**	Crozier, A., Y. Kamiya, G. Bishop, and T. Yokota. (2000). "Biosynthesis of Hormones and Elicitor Molecules" in *Biochemistry and Molecular Biology of Plants,* eds. B.B Buchanan, W. Gruissem, and R.L. Jones. Rockville, MD: American Society of Plant Physiologists, 850-929.<br>Nambara, E., and Marion-Poll, A. (2005). Abscisic Acid Biosynthesis and Catabolism. *Annu. Rev. Plant Biol.*, *56*, 165-185.	Cutler, S. R., Rodriguez, P. L., Finkelstein, R. R., and Abrams, S. R. (2010). Abscisic Acid: Emergence of a Core Signaling Network. *Annu. Rev. Plant Biol. 61*, 651-679.<br>Di Mauro, M. F., Iglesias, M. J., Arce, D. P., Valle, E. M., Arnold, R. B., Tsuda, K., Yamazaki, K., Casalongué, C.A., and Godoy, A. V. (2012). MBF1s Regulate ABA-Dependent Germination of Arabidopsis Seeds. *Plant Signal. Behav. 7*, 188-192.<br>Guo, J., Wang, J., Xi, L., Huang, W. D., Liang, J., and Chen, J. G. (2009). RACK1 is a Negative Regulator of ABA Responses in Arabidopsis. *J. Exp. Bot. 60*, 3819-3833.<br>Hubbard, K. E., Nishimura, N., Hitomi, K., Getzoff, E. D., and Schroeder, J. I. (2010). Early Abscisic Acid Signal Transduction Mechanisms: Newly Discovered Components and Newly Emerging Questions. *Gene Dev. 24*, 1695-1708.<br>Li, Z., Li, Z., Gao, X., Chinnusamy, V., Bressan, R., Wang, Z. X., Zhu, J.K., Wu, J.W., and Liu, D. (2012). ROP11 GTPase Negatively Regulates ABA Signaling by Protecting ABI1 Phosphatase Activity from Inhibition by the ABA receptor RCAR1/PYL9 in Arabidopsis. *J. Integr. Plant Biol*.<br>Quist, T. M., Sokolchik, I., Shi, H., Joly, R. J., Bressan, R. A., Maggio, A., Narsimhan, M., and Li, X. (2009). HOS3, an ELO-like Gene, Inhibits Effects of ABA and Implicates a S-1-P/Ceramide Control System for Abiotic Stress Responses in *Arabidopsis thaliana*. *Molecular Plant*, *2*, 138-151.<br>Raghavendra, A.S., Gonugunta, V.K., Christmann, A., and Grill, E. (2010). ABA Perception and Signalling. *Trends Plant Sci.* 15**,** 395-401.<br>Umezawa, T., Nakashima, K., Miyakawa, T., Kuromori, T., Tanokura, M., Shinozaki, K., and Yamaguchi-Shinozaki, K. (2010). Molecular Basis of the Core Regulatory Network in ABA Responses: Sensing, Signaling and Transport. *Plant Cell Physiol*. 51, 1821-1839.<br>Zhang, Y., Yang, C., Li, Y., Zheng, N., Chen, H., Zhao, Q., Gao, T., Guo, H., and Xie, Q. (2007). SDIR1 is a RING Finger E3 Ligase That Positively Regulates Stress-Responsive Abscisic Acid Signaling in Arabidopsis. *Plant Cell*, *19*, 1912-1929.
